# Pollinator competition and the contingency of nectar depletion during an early spring resource pulse

**DOI:** 10.1002/ece3.11531

**Published:** 2024-06-18

**Authors:** Douglas B. Sponsler, Murray Hamilton, Michael Wiesneth, Ingolf Steffan‐Dewenter

**Affiliations:** ^1^ Department of Animal Ecology and Tropical Biology, Biocenter University of Würzburg Würzburg Germany

**Keywords:** coexistence, competition, nectar, pollinator, trophic ecology

## Abstract

Concerns about competition between pollinators are predicated on the assumption of floral resource limitation. Floral resource limitation, however, is a complex phenomenon involving the interplay of resource production by plants, resource demand by pollinators, and exogenous factors—like weather conditions—that constrain both plants and pollinators. In this study, we examined nectar limitation during the mass flowering of rosaceous fruit trees in early spring. Our study was set in the same region as a previous study that found severe nectar limitation in summer grasslands. We used this seasonal contrast to evaluate two alternative hypotheses concerning the seasonal dynamics of floral resource limitation: either (H1) rates of resource production and consumption are matched through seasonal time to maintain a consistent degree of resource limitation, or (H2) a mismatch of high floral resource production and low pollinator activity in early spring creates a period of relaxed resource limitation that intensifies later in the year. We found generally much lower depletion in our spring study compared to the near 100% depletion found in the summer study, but depletion rates varied markedly through diel time and across sampling days, with afternoon depletion rates sometimes exceeding 80%. In some cases, there were also pronounced differences in depletion rates across simultaneously sampled floral species, indicating different degrees of nectar exploitation. These findings generally support the seasonal mismatch hypothesis (H2) but underscore the complex contingency of nectar depletion. The challenge of future work is to discern how the fluctuation of resource limitation across diel, inter‐diel, and seasonal time scales translates into population‐level outcomes for pollinators.

## Graphical abstract

Resource competition is predicated on resource limitation, which can be represented by the proxy of resource depletion rate. We used a pollinator exclusion experiment to estimate nectar depletion rates in an orchard meadow landscape during the early spring flowering of rosaceous trees. In contrast to our recent work in summer grasslands, we found relatively low depletion rates, suggesting that in spring, abiotic conditions (temperature, precipitation) are more important than competition as constraints on pollinator fitness.

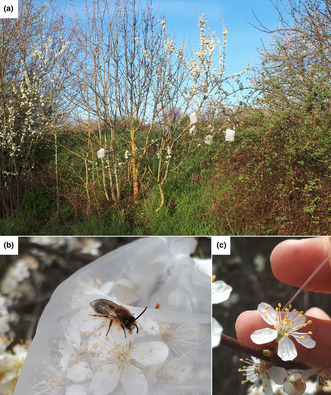

## 1 INTRODUCTION

Around the middle of March, the drab vernal forests of central Germany, not yet brightened with new foliage, became suddenly frosted with the white flowers of rosaceous fruit trees: first plum (*Prunus domestica*) and blackthorn (*P. spinosa*), then cherry (*Prunus* subg. *Cerasus*), pear (*Pyrus communis*), hawthorn (*Crataegus* spp.), and eventually apple (*Malus domestica*). Early in this progression, these fruit trees of the edge and understory are joined by the maples (*Acer* sp.) of the canopy, and this concert of floral phenology, packed densely into the first weeks of spring, is perhaps the greatest release of nectar and pollen experienced by flower‐visiting animals in the course of the year. This story plays out, with local variation, across much of the northern temperate zone (Couvillon et al., 2014; Requier et al., 2015; Sponsler et al., 2020; Wood et al., 2018).

Against such a backdrop of apparent plenty, the problem of floral resource competition among pollinators—once the inspiration (and often the frustration) of classical theoreticians, now the urgent business of applied conservationists (Sponsler, Iverson, & Steffan‐Dewenter, 2023)—would appear more nuanced than under the comparatively austere conditions that, in the same region, prevail later in summer (Sponsler et al., 2024). In the sea of flowers comprised by a blackthorn hedge or a forest margin lined with plum trees, is it plausible that nectar and pollen are limiting resources for which pollinators must compete?

It is a question that should not be answered hastily. The visible abundance of flowers is not necessarily a reliable proxy for the production of floral resources (Zimmerman & Pleasants, 1982), since the latter can vary dramatically not only across floral species but among plants of the same species, flowers of the same plant, and even nectaries of the same flower (Herrera et al., 2006). It is, moreover, a perennial error among animal ecologists to overlook the agency of plants, which can, over both evolutionary and behavioral time scales, tune the release of floral rewards to modulate pollinator behavior and avoid unnecessary energy expenditure (Mu et al., 2014; Pyke, 1991; Ratnieks & Balfour, 2021). The seasonal population dynamics of pollinator communities might, in turn, conform to the seasonal dynamics of floral resource availability such that the ratio of production and consumption remains fairly constant throughout the year (Pleasants, 1981). The tight correlation between seasonal patterns of floral and pollinator diversity evident in British phenological records (Balfour et al., 2018) could be interpreted as evidence in favor of this claim.

Yet there is also reason to suspect that the early spring flush of floral abundance driven by flowering trees could be a period of true trophic surplus and competitive release for pollinators. At the onset of spring, insect populations are constrained by temperature, irrespective of food availability; many solitary species have not yet emerged, and eusocial species with annual life cycles (e.g. bumble bees, social sweat bees) have not yet built up large colonies. Even the perennial colonies of honey bees, while already containing thousands of workers, are a fraction of the peak size they will attain by midsummer. Moreover, due to the poikilothermic nature of insects, the low temperatures of early spring limit not only the abundance of pollinators but also their *activity*. On a sufficiently cold morning, the effective pollinator population is always zero, and the incremental increase in temperature throughout the day presumably releases pollinator species in accordance with their respective cold tolerances. Thus, based on strict physiological constraints, the flowering of trees in early spring coincides with relatively low pollinator density. Under such conditions, pollinators might not only enjoy a relatively high flower‐to‐forager ratio but also benefit from selection pressure on plants to compete with one another for a limiting supply of pollination visits by investing more energy in the production of floral rewards (Mu et al., 2014; Ratnieks & Balfour, 2021). A growing body of empirical evidence supports the idea that floral resource availability is uneven through seasonal time and, at least in northern temperate regions, tends to be higher in spring than in summer (Bishop et al., 2024; Couvillon et al., 2014; Garbuzov et al., 2020; Sponsler et al., 2020; Timberlake et al., 2019).

These lines of reasoning can be formulated as alternative hypotheses concerning competition between pollinators for floral resources: either (H1) the proportionality between the production and consumption of floral resources is stabilized by the joint phenology and behavior of plant and pollinator communities, thus maintaining consistent competitive intensity, or (H2) seasonal asynchrony between the production and consumption of floral resources causes low competition intensity in the spring that gives way to higher competition intensity later in the year. These two hypotheses imply very different understandings of plant‐pollinator ecology. Under H1, pollinators that emerge at different times of year all experience a similar degree of competitive intensity, and pollinators with long active seasons (e.g. bumble bees, honey bees) experience similarly competitive conditions throughout their whole annual life cycle. If the stable competition intensity predicted by H1 is high, then competition should be a strong driver of pollinator community assembly. Moreover, stably high competition among pollinators for limited floral resources would imply the opposite condition for plants, which should (on average) enjoy a stable surplus of pollinator visitation (Sponsler, Iverson, & Steffan‐Dewenter, 2023). Alternatively, if competition is stably low, then competition should not be an important driver of pollinator community assembly, but plants would be expected, conversely, to compete for a limiting supply of pollinator visits. Under H2, however, the importance of competition as a driver of community assembly would be seasonally variable, and pollinator species with long active seasons would have to be able to cope with a mid‐season transition in competition intensity. Similarly, the plants that drive the early spring resource pulse would be pollinator‐limited, while those that bloom later in the year would benefit from a surplus of visits from a population of food‐limited pollinators.

Our previous work in central Germany found evidence of high competition intensity in midsummer grasslands (Sponsler et al., 2024). In the present study, we replicated our competition assay in the same study region during the mass flowering of plum, cherry, and pear trees in early spring. H1 predicts that nectar depletion rates in spring should be comparable to the high rates seen in midsummer, while H2 predicts substantially lower nectar depletion rates in spring. We test these predictions and interpret our results with respect to the question of the relative importance of trophic and nontrophic constraints on pollinator populations.

## 2 METHODS

### 2.1 Study sites and sampling regime

Following Sponsler et al. (2024), we conducted a pollinator exclusion experiment to estimate nectar depletion rate, which can be treated as a proxy for competition intensity (Sponsler, Iverson, & Steffan‐Dewenter, 2023). The depletion rate can be understood as the proportional difference between the *realized* rate of nectar reward (represented by the nectar standing crop) experienced by the average forager in the observed pollinator community and the *potential* rate of nectar reward experienced by a hypothetical lone forager collecting nectar in the same landscape (i.e. a forager that would encounter only unvisited flowers).

Our study was set near the city of Würzburg in central Germany, in a landscape consisting of early‐successional forest and seminatural orchard meadows, bordered by agricultural fields to the south, a university campus to the north and west, and a residential neighborhood to the east (Figure S1). The woody vegetation of the landscape was rich in rosaceous fruit trees, including plum (*Prunus domestica*), blackthorn (*P. spinosa*), cherry (*Prunus* subg. Cerasus), and pear (*Pyrus communis*)—hereafter referred to by their common names—which we included in our experiment according to their respective flowering phenologies. Maple trees were also abundant and in bloom during our study, but we did not sample them because their high canopies prevented ready access to their flowers. Hawthorn (*Crataegus* spp.) and apple (*Malus domestica*) were common in the landscape but did not bloom until after our sampling was concluded. This composition of woody vegetation is typical for the region, but our study site was enriched in plum and pear tree density because of a legacy of former cultivation in the vicinity, and the trees we sampled probably include both surviving cultivated trees and those seeded adventitiously after the land was abandoned. Of the four cherry trees we sampled, three were found on the university campus and were evidently planted as amenities, while the southernmost tree was either wild or a survivor of the former orchards. All the blackthorn shrubs in the landscape were presumably wild, as this species occurs ubiquitously in disturbed habitats in our region. Due to the presence of research apiaries associated with the university, local honey bee density was exceptionally high, with at least 55 colonies located within 500 m of our sampling area.

Sampling began on March 20th, 2023, and continued for 1 month, concluding on April 21st. Sampling was conducted opportunistically within that interval on days without precipitation during sampling hours and with midday temperatures above 10°C, resulting in a total of nine sampling days.

**FIGURE 1:**
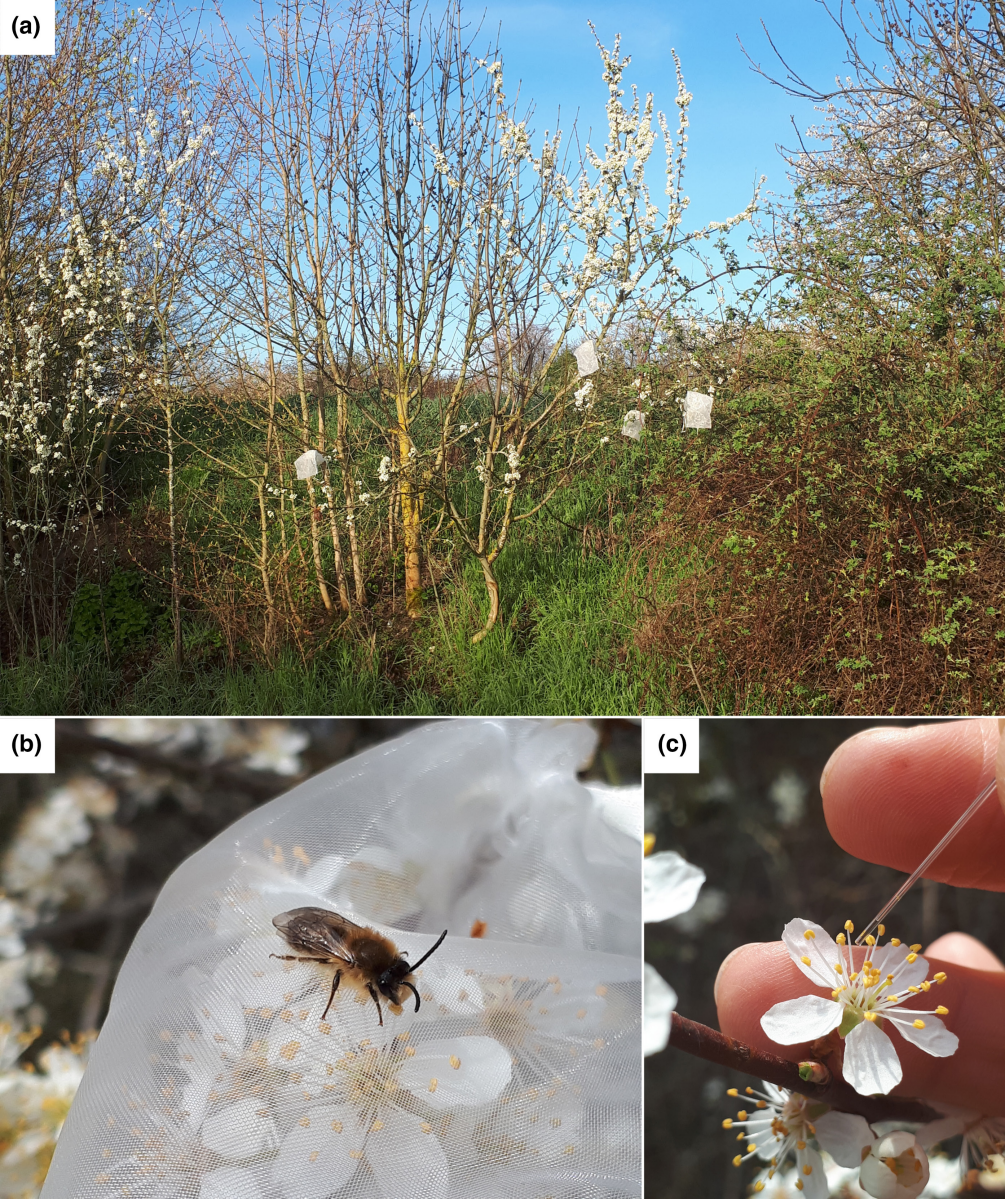
Illustration of sampling methods. Trees selected for sampling were bagged early in the morning, before the onset of pollinator activity (a). Bags were constructed of a fine synthetic mesh that effectively excluded insect visitors while allowing air to flow relatively unobstructed (b). Nectar was sampled by probing each flower with a microcapillary tube (c); in this image, a small volume of nectar is visible below the meniscus near the distal end of the tube.

On each day of sampling, five mesh bags, large enough to cover the distal end of a tree branch with a length of about 20 cm, were installed on each tree selected to be sampled (Figure 1a). Bagging was done before 8:00, while temperatures were cool enough to preclude pollinator activity. Bags were constructed of synthetic fabric similar to mosquito netting (Figure 1b), which has a minimal influence on the microclimate of the flower (Kearns et al., 1993). Sampling was conducted at intervals beginning at 9:00 and repeated every 2 h until 15:00 unless interrupted by rain, and on one day we did an additional round at 17:00. In each round, we sampled five bagged flowers and five open flowers from each tree. Since the bags we used covered multiple flowers, only one bag was removed per sampling round, leaving the remaining bagged flowers covered to prevent extraneous visitation during sampling. Both bagged and open flowers were chosen according to the criteria of having an intact stigma (i.e. not senescent) and actively dehiscing anthers. The second of these criteria was adopted after the second day of sampling, when we noticed that nectar yields appeared to be higher in flowers that were visibly dehiscing. Open flowers were selected in close proximity to bagged flowers to minimize any confounding effects of within‐tree spatial variation in nectar availability. Because blackthorn occurs in dense hedges in which individual shrubs cannot readily be distinguished, we sampled one pair of flowers (i.e. one bagged, one open) from a given branch and then repeated this for a total of five branches separated by enough distance along the hedge that we could be sure they belonged to different shrubs. Nectar was sampled using 0.5, 1.0, or 5 μL microcapillary tubes (Hirschmann, Eberstadt; Figure 1c), depending on the expected volume of nectar. When necessary, multiple tubes were used to extract the full volume of nectar from a given flower. The concave shape of the nectary in cherry flowers required that flowers be split cross‐sectionally to access nectar (Figure S2).

We sampled 3–4 trees per day, except for the first day of sampling in which only one tree could be sampled, and with the exception of blackthorn, due to the methodological accommodation explained above that required the sampling of five individual trees per sampling round. In total, we sampled 12 plum trees, 27 blackthorn shrubs, four cherry trees, and four pear trees, including a total of 621 bagged flowers and 627 open flowers. We also obtained hourly temperature and precipitation data from a public weather station located about 3.2 km from our study area. Trees were selected opportunistically in accordance with phenological progression; that is, we selected trees that were in full bloom and sampled them until their flowers began to appear senescent. This resulted in a taxonomic sequence that began with plum, then proceeded to blackthorn, then a second wave of plum accompanied by cherry, and finally pear (Figure S3). Note that the blooming of blackthorn actually extended well beyond the period during which we sampled it, but we opted to prioritize the sampling of plum and cherry once these became available.

On April 14 and 21, we measured the sugar concentration of sampled nectar with a handheld refractometer (Bellingham & Stanley, UK) whenever we collected a sufficient volume to ob a reliable reading. For concentration measurements, we pooled the five nectar samples collected from each treatment (i.e. bagged vs. open) for a given tree in a given sampling round. Because bagged flowers typically yield a higher volume of nectar due to the exclusion of insect visitors, we more often obtained concentration readings from the bagged treatment. On April 21st, we noted that, after a cool night with light precipitation, flowers were covered with a heavy coat of dew. This provided an opportunity to explore the effect of dew on nectar concentration. Similarly, on March 22, flowers were too wet to sample reliably at the 9:00 sampling round due to early morning rain; on this date, though, we did not have a refractometer prepared to measure nectar concentration.

### 2.2 Data analysis

For the purposes of our analysis, we defined resource depletion rate (*D*) as the proportional difference between the mean resource volume of bagged (*V*_bagged_) and open (*V*_open_) flowers:

(1) $$D=1-\frac{V_{\text{open}}}{V_{\text{bagged}}}$$

To respect the hierarchical structure of our sampling, in which observations were grouped within trees, species, sampling rounds, and days, we estimated *V*_bagged_ and *V*_open_ using a hierarchical Bayesian regression model. We preprocessed our data by normalizing our nectar measurements and dividing each reading by the mean volume of the bagged flowers within each species, round, and date. This facilitated prior selection and dampened variation extraneous to the task of comparing relative volumes across treatments. Since volume is continuously non‐negative but includes zeros, we used a hurdle‐gamma model consisting of gamma and binomial submodels. For both the gamma and binomial submodels, treatment and round were specified as interacting fixed effects, and tree, species, and date were specified as varying (i.e. “random”) slope and intercept effects. All models were assigned regularizing and weakly informative priors (Wesner & Pomeranz, 2021), following the workflow described by Gabry et al. (2019). After validating our model, we calculated *D* by applying (1) to the posterior predictive distributions of *V*_bagged_ and *V*_open_. We then censored the posterior predictive distribution by converting all negative values to zero, since (1) produces extremely high negative values for posterior draws in which the volume of the open treatment is higher than the bagged treatment, and there is no meaning to the idea of “negative” depletion. Importantly, the posterior distribution of (censored) *D* is the probability distribution for the *mean* depletion rate, not the expected distribution of *observed* depletion rates. Thus, all references to “depletion rate” hereafter refer to the expected mean depletion rate inferred by our model.

We mapped our study area (Figure S1) using QGIS (QGIS Development Team, 2023). All other data operations were done in R v. 4.3.2 (R Core Team, 2023). Data handling was performed with the tidyverse package suite (Wickham et al., 2019). We implemented our regression model with Stan (Stan Development Team, 2022), accessed via brms (Bürkner, 2017). Model predictions were extracted using tidybayes (Kay, 2021b), and models were visualized using ggplot2 (Wickham, 2016), see (Lüdecke et al., 2021), and ggdist (Kay, 2021a). A complete workflow, including all codes needed to reproduce and validate our models is provided in Appendices S1 and S2.

## 3 RESULTS AND DISCUSSION

**FIGURE 2:**
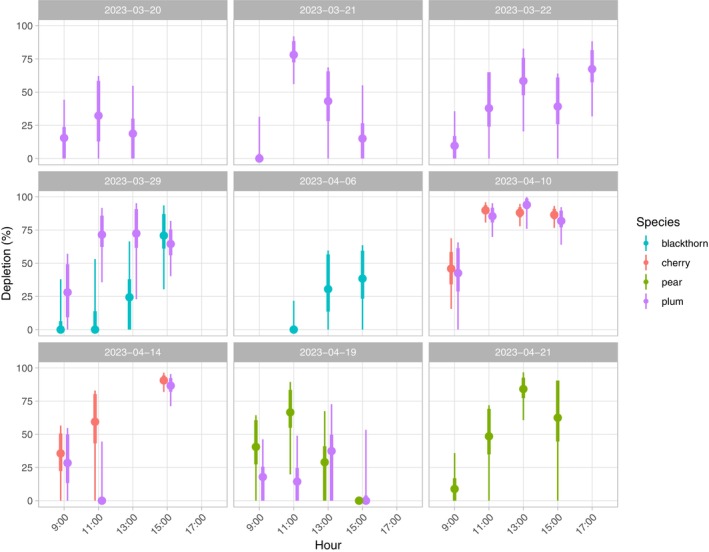
Mean depletion rate by species, day, and hour (i.e. sampling round). Estimated mean depletion rates are visualized with point‐interval plots, with the inner and outer intervals representing 66% and 95% of the posterior probability distribution, respectively.

Nectar depletion rates in our study were highly variable but usually much lower than the near 100% depletion observed in nearby grasslands during the preceding summer (Sponsler et al., 2024), lending tentative support to the hypothesis (H2) that seasonal asynchrony between nectar production and nectar consumption creates a transition from less competitive conditions in spring to more competitive conditions in summer (Figure 2). Nevertheless, estimated depletion rates exceeded 80% in some samples on April 10, 14, and 21, demonstrating that nectar limitation can be significant even under the conditions of apparent floral abundance during the mass flowering of rosaceous trees in early spring.

The most pronounced pattern in our data is the diel variation in depletion rate. Depletion rates were consistently lowest during the first (9:00) sampling round and generally increased throughout the day, with the highest rates of depletion usually seen during the 13:00 or 15:00 sampling rounds. Studies in the tropics and subtropics have found that the standing crop of nectar is often reduced to a minimum within a few hours of the onset of foraging (Brown et al., 1981; Collins et al., 1984; Roubik & Buchmann, 1984). Our results show a broadly similar pattern, though with a somewhat later peak of nectar depletion, likely due to the dampening effect of cool morning temperatures on pollinator activity. Two mutually compatible explanations could account for this pattern. First, the gradual increase in air temperature throughout the day probably causes a corresponding increase in pollinator activity and floral visitation rate. Second, increasing depletion rates throughout the day would be expected simply as a cumulative result of sustained foraging activity, so long as the rate of nectar secretion is lower than the rate of nectar removal. It is worth nothing that since honey bees are especially averse to foraging at low temperatures, pollinator species with higher cold tolerance may escape competition by foraging early in the day (Araújo et al., 2022; Tepedino, 1981). Indeed, we noted anecdotally that pollinator visitation during the earlier sampling rounds tended to be dominated by bees of the genera *Osmia*, *Colletes*, and *Andrena*, with honey bees becoming the most abundant visitors later in the day.

Notably, depletion rates sometimes differed across species that were sampled simultaneously. For example, on March 29th, depletion was much higher in plum than in blackthorn for the first three sampling rounds, before the two species reached approximately equal depletion rates during the 15:00 sampling round. Similarly, depletion rates were higher in pear than in plum for the first two sampling rounds of April 19th. In the case of blackthorn, we observed that visiting honey bees almost always carried pollen loads in their corbiculae, so it is possible that nectar depletion rates were low because blackthorn was visited primarily for its pollen reward. Though we did not measure pollen depletion, it was visually obvious that the anthers of blackthorn flowers were progressively stripped of pollen throughout the day, suggesting that pollen may have been limiting despite low nectar depletion rates. It is also possible that differences in nectar quality (e.g. sugar concentration), flower cover, or spatial configuration in the landscape could cause some species to be more heavily exploited by nectar foragers than others. For example, in a study of co‐flowering apple and pear trees, Quinet et al. (2016) found that pear flowers were visited primarily for pollen while pollinators focused their nectar foraging on apples, a pattern the authors attribute to the higher sugar concentration of apple nectar.

One of the defining features of early spring in our study region, as in much of the temperate zone, is fluctuating temperature and frequent precipitation. For poikilothermic organisms like insect pollinators, these vagaries of temperature and precipitation impose strict limits on activity. While pollinator species vary in their tolerance of cold and precipitation, honey bees forage very little below 10°C or in any appreciable precipitation (Abou‐Shaara et al., 2017). Based on these criteria, only about 40% of daylight hours during the period of our study qualified as suitable foraging conditions (Figure S4). This strict temperature limitation is probably one of the reasons we observed lower depletion rates than those seen in our study region in the summer of the preceding year (Sponsler et al., 2024), when warm and dry conditions allowed almost uninterrupted foraging during daylight hours. While drawing conclusions about the role of weather and temperature in modulating resource exploitation and competition between pollinators is beyond the scope of our study, the hypothesis that pollinators are generally limited by weather in spring and by food limitation in summer is consistent with the more general theory, originally advanced by Kropotkin (1902) and revived by more recent theoretical (Bertness and Callaway, 1994) and empirical (Classen et al., 2020) work, that there exists a negative relationship between environmental adversity and competitive intensity.

Importantly, we conducted this study under conditions of extremely high honey bee density, with at least 55 colonies in the vicinity of the trees we sampled. If we say conservatively that each of these colonies had an average of 5000 workers tasked with foraging, this foraging force of 275,000 probably dwarfed the number of wild pollinators concurrently gathering nectar in the landscape. This raises the question of what depletion rates *would* have been under the average density (in Germany) of 1–3 managed honey bee colonies per square kilometer (De la Rúa et al., 2009) or, hypothetically, under the total absence of managed honey bees. It seems likely that under these alternative scenarios, depletion rates would have been much lower than the ones we observed. The fact that we observed relatively low rates of depletion despite unnaturally high honey bee densities strengthens the evidence in favor of the seasonal asynchrony hypothesis (H2) of pollinator competition. It is also worth asking whether a disproportionate volume of honey bee foraging can “tip” spring landscapes into competitive states that would otherwise not be expected until later in the year. Such an effect could pose a challenge to spring‐active pollinator species (including the foundress queens of bumble bee colonies) if they are adapted to foraging under relatively noncompetitive conditions.

**FIGURE 3:**
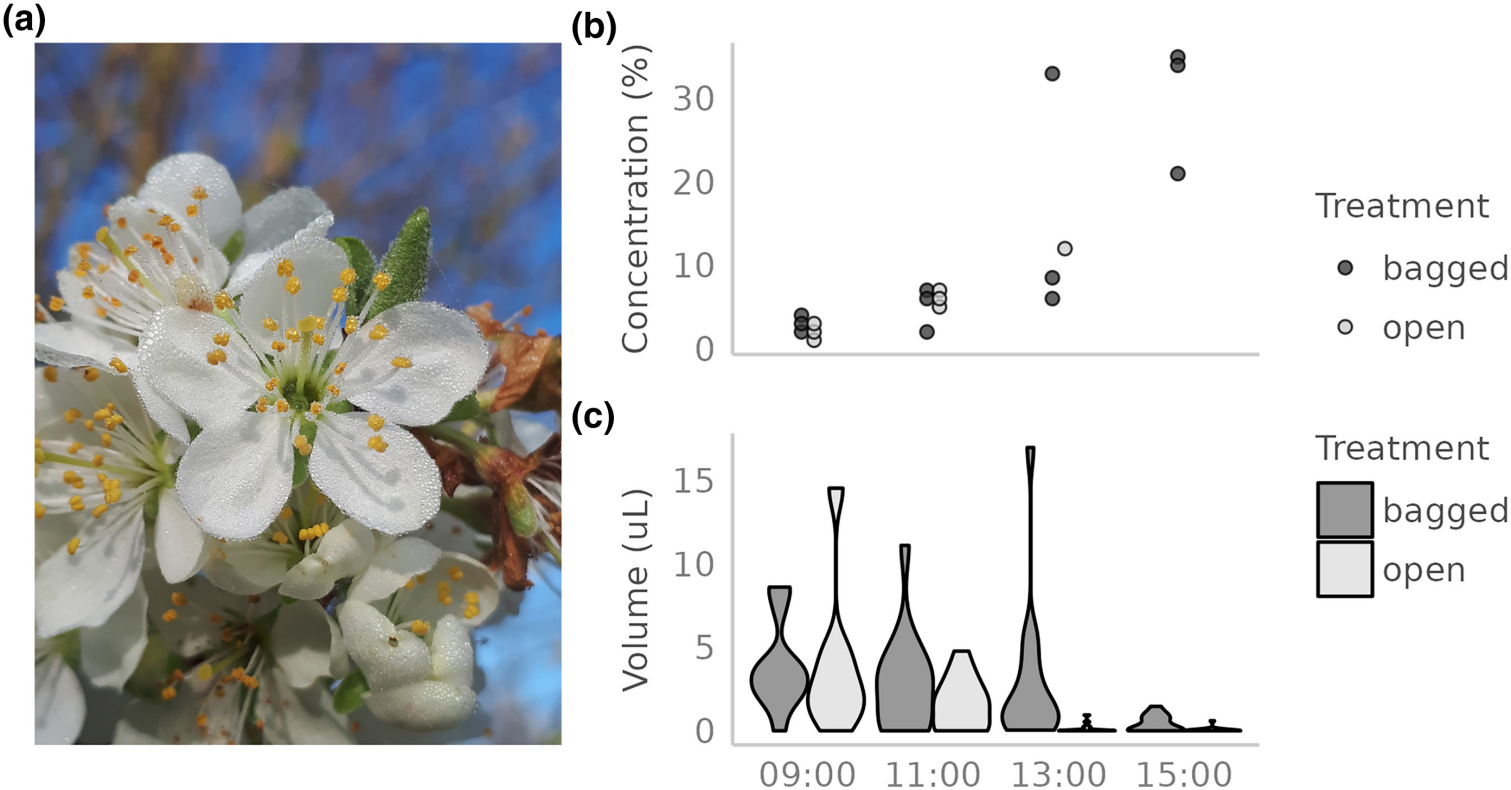
Dilution of *Pyrus communis* (pear) nectar by dew on April 21st. The photograph (a), though depicting a *P. domestica* flower on April 14th, illustrates the appearance of dew on a flower in the early morning. Throughout the day, presumably due to the evaporation of dew and the continued secretion of nectar, sugar concentration increased from near 1% to near 30% (b), with a corresponding decrease in volume (c). Notably, the pattern was similar across bagged and open treatments, suggesting that the decrease in volume was driven by evaporation (or resorption) rather than pollinator foraging.

While seemingly tangential to our primary question of resource depletion and competition, the phenomenon of nectar dilution deserves consideration. As described earlier, we noticed on the morning of April 21st that the pear flowers we were sampling were coated with a heavy layer of dew (Figure 3a). Throughout the day, we observed a gradual increase in nectar sugar (sucrose‐equivalent) concentration, beginning at <5% at 9:00 and peaking at 20–35% by 15:00 (Figure 3b). Over the same time interval, nectar volume in open flowers steadily declined to near emptiness (Figure 3c). Interestingly, nectar volume was also very low in *bagged* flowers by the end of the day, suggesting that the decline in volume in both bagged and open flowers was driven in part by processes other than nectar removal by pollinators, such as evaporation or resorption. The inverse correlation of volume and concentration indicates that the high nectar volume seen in earlier samples was probably driven in large part by the dilution of nectar with dew. Throughout the day, presumably due to the evaporation of water and the continued secretion of nectar sugars, sugar concentration increased to levels in the range expected for pear flowers (Pamminger et al., 2019). Subtler manifestations of this phenomenon may have contributed to the general pattern of diel increase in depletion rate (Figure 2), since increasing nectar concentrations would be expected to attract more intense visitation. The most salient observation, though, is simply that morning dew can dilute the standing crop of nectar to near‐zero sugar concentrations, rendering them nutritionally useless to pollinators. Under such conditions, flowers with morphologies that protect nectaries from the incursion of dew would be the only flowers worth visiting.

We recommend caution, of course, in generalizing our findings beyond the local context of our case study. The fundamental processes behind floral resource limitation—the joint dynamics of resource production and consumption—are independent of context, but the relative strengths of these processes, their modulation by exogenous drivers, and their ultimate consequences for fitness and coexistence can be expected to vary markedly across space, time, and the functional trait space of plan‐pollinator communities (Sponsler, Iverson, & Steffan‐Dewenter, 2023). Our results, moreover, pertain only to nectar limitation; the severity and contingency of pollen depletion and the degree to which patterns of pollen depletion mirror those of nectar depletion remain an open question, though Harris et al. (2023) have recently reported similar depletion rates for nectar (41%) and pollen (56%) during the mass flowering of English ivy (*Hedera helix*).

## 4 CONCLUSION

Broadly speaking, our study, interpreted in conjunction with Sponsler et al. (2024), supports the hypothesis (H2) that seasonal asynchrony between the production and consumption of floral resources causes pollinators to experience relatively low nectar limitation in the spring and higher nectar limitation later in the year, at least in the local context of our study area. This pattern is probably driven not only by the relatively high ratio of floral abundance to pollinator abundance during the mass bloom of fruit trees but also by the erratic weather conditions of early spring that, through cold and precipitation, impose strict limits on pollinator activity.

Nevertheless, our results tell a story of nectar limitation that is more complex than a simple transition from spring surplus to summer scarcity. Behind moderate overall rates of depletion, we observed strong temporal variation at diel and inter‐diel scales. Even on days when average depletion is low, depletion rates in the afternoon can reach levels that probably have fitness consequences. This elusive endpoint of fitness is the final criterion of competition, and understanding how the dueling patterns of floral resource dynamics and pollinator behavior translate into population‐level outcomes is perhaps the most daunting challenge confronting a trophic‐ecological approach to plant‐pollinator interactions (Sponsler, Iverson, & Steffan‐Dewenter, 2023).

## AUTHOR CONTRIBUTIONS

**Douglas B. Sponsler:** Conceptualization (lead); data curation (lead); formal analysis (lead); investigation (lead); methodology (lead); project administration (equal); software (lead); supervision (equal); visualization (lead); writing – original draft (lead); writing – review and editing (lead). **Murray Hamilton:** Data curation (supporting); investigation (equal); methodology (supporting); writing – review and editing (supporting). **Michael Wiesneth:** Data curation (supporting); investigation (equal); methodology (supporting); writing – review and editing (supporting). **Ingolf Steffan‐Dewenter:** Conceptualization (supporting); funding acquisition (lead); project administration (lead); resources (lead); supervision (equal); writing – review and editing (supporting).

## CONFLICT OF INTEREST STATEMENT

The authors declare no conflicts of interest.

## Supporting information


Appendix S1.

## Data Availability

All data described in this paper are included as supplemental material, together with a fully reproducible analytical pipeline included in Appendices S1 and S2. All data and codes are also available on Dryad: https://doi.org/10.5061/dryad.573n5tbgg.
