# Supplementary material for: Pollinator competition and the contingency of nectar depletion during an early spring resource pulse
Source: Ecol Evol. 2024 Jun 18;14(6):e11531. doi: 10.1002/ece3.11531 (PMC11183943; doi:10.1002/ece3.11531)
Supplement: Supplementary file 1 — Appendix S1. [file ECE3-14-e11531-s001.zip › ece311531-sup-0006-AppendixS6.docx]

# Pollinator competition and the contingency of nectar depletion during an early spring resource pulse

Appendix 1: Model fitting and validation

Douglas B. Sponsler* Murray Hamilton Michael Wiesneth Ingolf Steffan-Dewenter

2024-05-23

## Contents

[Summary](file:///\\172.24.191.130\Straive_E3\Journals\WILEY_JNLS\RAW\ECE3\ECE3_11531\FPP\doc\ECE3_11531_AM.docx#_bookmark0) 1

[Packages](file:///\\172.24.191.130\Straive_E3\Journals\WILEY_JNLS\RAW\ECE3\ECE3_11531\FPP\doc\ECE3_11531_AM.docx#_bookmark1) 3

[Load data](file:///\\172.24.191.130\Straive_E3\Journals\WILEY_JNLS\RAW\ECE3\ECE3_11531\FPP\doc\ECE3_11531_AM.docx#_bookmark2) 3

[Priors](file:///\\172.24.191.130\Straive_E3\Journals\WILEY_JNLS\RAW\ECE3\ECE3_11531\FPP\doc\ECE3_11531_AM.docx#_bookmark3) 3

[Gamma global intercept](file:///\\172.24.191.130\Straive_E3\Journals\WILEY_JNLS\RAW\ECE3\ECE3_11531\FPP\doc\ECE3_11531_AM.docx#_bookmark4) 3

[Gamma global treatment](file:///\\172.24.191.130\Straive_E3\Journals\WILEY_JNLS\RAW\ECE3\ECE3_11531\FPP\doc\ECE3_11531_AM.docx#_bookmark5) 4

[Gamma groupwise standard deviation](file:///\\172.24.191.130\Straive_E3\Journals\WILEY_JNLS\RAW\ECE3\ECE3_11531\FPP\doc\ECE3_11531_AM.docx#_bookmark6) 5

[Binomial global intercept](file:///\\172.24.191.130\Straive_E3\Journals\WILEY_JNLS\RAW\ECE3\ECE3_11531\FPP\doc\ECE3_11531_AM.docx#_bookmark7) 6

[Binomial global treatment](file:///\\172.24.191.130\Straive_E3\Journals\WILEY_JNLS\RAW\ECE3\ECE3_11531\FPP\doc\ECE3_11531_AM.docx#_bookmark8) 7

[Binomial groupwise standard deviation](file:///\\172.24.191.130\Straive_E3\Journals\WILEY_JNLS\RAW\ECE3\ECE3_11531\FPP\doc\ECE3_11531_AM.docx#_bookmark9) 8

[Call prior set](file:///\\172.24.191.130\Straive_E3\Journals\WILEY_JNLS\RAW\ECE3\ECE3_11531\FPP\doc\ECE3_11531_AM.docx#_bookmark10) 9

[Prior-predictive check](file:///\\172.24.191.130\Straive_E3\Journals\WILEY_JNLS\RAW\ECE3\ECE3_11531\FPP\doc\ECE3_11531_AM.docx#_bookmark11) 11

[Update model with data](file:///\\172.24.191.130\Straive_E3\Journals\WILEY_JNLS\RAW\ECE3\ECE3_11531\FPP\doc\ECE3_11531_AM.docx#_bookmark12) 12

[Validate model](file:///\\172.24.191.130\Straive_E3\Journals\WILEY_JNLS\RAW\ECE3\ECE3_11531\FPP\doc\ECE3_11531_AM.docx#_bookmark13) 12

[Posterior predictive check](file:///\\172.24.191.130\Straive_E3\Journals\WILEY_JNLS\RAW\ECE3\ECE3_11531\FPP\doc\ECE3_11531_AM.docx#_bookmark14) 12

[Check influence of priors](file:///\\172.24.191.130\Straive_E3\Journals\WILEY_JNLS\RAW\ECE3\ECE3_11531\FPP\doc\ECE3_11531_AM.docx#_bookmark15) 13

## Summary

Our analytical objective was to calculate floral resource depletion rate (*D*), which we defined as the proportional difference between the mean resource volume of bagged (*V_bagged_*) and open (*V_open_*) flowers:

*Vopen*

= 1

*D −*

*Vbagged*

(1)

Simply using the empirical estimates for *V_bagged_* and *V_open_*, however, would ignore the dependencies in our sampling structure, thereby underestimating the uncertainty in our estimate of *D*. Specifically, our measurements of *V_bagged_* and *V_open_* are nested within individual trees, which in turn are nested within species, sampling rounds, and sampling dates.

To account for this sampling structure in a principled way, we estimated *V_bagged_* and *V_open_* using hierarchical Bayesian regression models with resource volume as the response variable, *treatment* (bagged vs. open) and *round* a constant (i.e. “fixed”) effects, and *tree* , *species*, and *date* as varying (i.e. “random”) slope and intercept effects, following the nomenclature recommended by Gelman (2005). Since our measurements of resource volume are continuously non-negative but include zeros, we specified hurdle-gamma models consisting of gamma and binomial submodels, each with the effects structure described above. After validating our models, we calculate *D* by applying **(1)** to the posterior predictive distributions of *V_bagged_* and *V_open_* (see Appendix 2).

Prior to modeling, we normalized the nectar measurements by dividing each reading by the mean reading of the bagged flowers within each tree, species, and round, and date. This facilitated prior selection and dampened variation extraneous to the task of comparing relative volumes across treatments. All models were specified with weakly informative priors (Wesner and Pomeranz 2021) following the modeling workflow describe by Gabry et al. (2019).

This appendix provides the workflow and output for the modeling procedure described above. As noted in the relevant section headers, time-consuming steps have been commented out and their output loaded from file. These files are included with the supplemental material of our manuscript. Alternatively, users can simply un-comment the relevant code blocks and reproduce the files, but this will increase the run time of the script.

## Packages Load data

nectar <- **read_rds**("../data/processed/hubland_nectar.rds")

**Priors**

These are log-link hurdle-Gamma models, so we need to think about the Gamma submodel priors on the log scale and the binomial submodel priors on the logit scale.

### Gamma global intercept

This is the mean relative nectar volume when all predictors are at their “means.” For factors, of course, you can’t take a mean, so we need to think about the mean relative nectar volume for the baseline of treatment, which is bagged. Since I normalized the data, this mean will be very close to 1 on the response scale, and therefore very close to 0 on the link scale. Importantly, though, I normalized nectar volume within each date, round, and tree by dividing all *nonzero* measurements by the mean, since dividing zero throws an error. What this means is that the mean that I divide through by will be lower (because it was calculate including the zeros) than the mean of the non-zero volumes, which will make the estimated intercept higher than 1. We’ll go with N(0, 0.25).

**tibble**(N_0_0.25 = **rnorm**(1e5, 0, 0.25),

N_0_0.10 = **rnorm**(1e5, 0, 0.10),

N_0_0.05 = **rnorm**(1e5, 0, 0.05),

N_0_0.25.res = **exp**(N_0_0.25), N_0_0.10.res = **exp**(N_0_0.10), N_0_0.05.res = **exp**(N_0_0.05)) **%>%**

**pivot_longer**(**everything**(), names_to = "dist") **%>%**

**mutate**(type = **if_else**(**grepl**(".res", dist), "response", "link")) **%>%**

**ggplot**(**aes**(value, fill = type, color = type)) **+ geom_density**() **+**

*#geom_histogram() +*

**stat_pointinterval**(.width = **c**(0.5, 0.75, .99),

color = "black") **+**

**ylim**(**c**(0, NA)) **+**

**labs**(x = "Value", y = "Probability density", fill = "Scale", color = "Scale") **+ facet_wrap**(**~**dist, scales = "free", ncol = 2) **+**

**scale_y_continuous**(NULL, breaks = NULL)


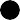


**N_0_0.05**


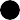


**N_0_0.05.res**

−0.2 −0.1 0.0 0.1 0.2 0.8 0.9 1.0 1.1 1.2


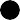


**N_0_0.10**


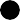


**N_0_0.10.res**

−0.25 0.00 0.25 0.8 1.0 1.2 1.4 1.6

#### Scale


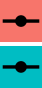
link response


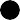


**N_0_0.25**


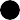


**N_0_0.25.res**

−1.0 −0.5 0.0 0.5 1.0 1 2 3

Value

### Gamma global treatment

The effect of treatment gets added (on the link scale) to the global intercept. Since this is the effect of going from treatment = bagged to treatment = open, it is reasonable to make this prior informatively negative.

Assuming an intercept of 0 (1 on response scale), a treatment effect of - 0.5 would result in open flowers having 60% [exp(0 - 0.5) = 0.6] nectar volume relative to bagged flowers. An effect of -1 would be 37%. An effect of -3 would be 5%. And -5 would be less than 1%.

On the other end, and effect of positive 0.5 would be 165%, and an effect of 1 would be 207%.

I need this prior to be tight on the positive side but generous on the negative side to allow for very high rates of depletion. N(-1, 1) looks reasonable.

**tibble**(N_n1.5_1.0 = **rnorm**(1e5, **-**1.5, 1),

N_n2.0_1.0 = **rnorm**(1e5, **-**2, 1),

N_n1.0_0.85 = **rnorm**(1e5, **-**1, 0.85),

N_n1.5_1.0.res = **exp**(0 **+** N_n1.5_1.0), N_n2.0_1.0.res = **exp**(0 **+** N_n2.0_1.0), N_n1.0_0.85.res = **exp**(0 **+** N_n1.0_0.85)) **%>%**

**pivot_longer**(**everything**(), names_to = "dist") **%>%**

**mutate**(type = **if_else**(**grepl**(".res", dist), "response", "link")) **%>%**

**ggplot**(**aes**(value, fill = type, color = type)) **+**

*#geom_density() +*

**stat_pointinterval**(.width = **c**(0.5, 0.75, .99),

color = "black") **+**

**ylim**(**c**(0, NA)) **+**

**labs**(x = "Value", y = "Probability density", fill = "Scale", color = "Scale") **+ facet_wrap**(**~**dist, scales = "free", ncol = 2) **+**

**scale_y_continuous**(NULL, breaks = NULL)


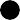


**N_n1.0_0.85**

| **N_n1.0_0.85.res** | | | | | | | | |
| --- | --- | --- | --- | --- | --- | --- | --- | --- |
|  |  |  |  |  |  |  |  |  |
|  |  |  |  |  |  |  |  |  |


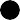

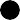

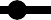
−3 −2 −1 0 1 0 1 2 3

| **N_n1.5_1.0** | | | | | | | | | | | |
| --- | --- | --- | --- | --- | --- | --- | --- | --- | --- | --- | --- |
|  |  |  |  |  |  |  |  |  |  |  |  |
|  |  |  |  |  |  |  |  |  |  |  |  |

| **N_n1.5_1.0.res** | | | | | | |
| --- | --- | --- | --- | --- | --- | --- |
|  |  |  |  |  |  |  |
|  |  |  |  |  |  |  |

−4 −3 −2 −1 0 1 0 1 2 3

| **N_n2.0_1.0** | | | | | | | | | | | |
| --- | --- | --- | --- | --- | --- | --- | --- | --- | --- | --- | --- |
|  |  |  |  |  |  |  |  |  |  |  |  |
|  |  |  |  |  |  |  |  |  |  |  |  |

| **N_n2.0_1.0.res** | | | | | | | | |
| --- | --- | --- | --- | --- | --- | --- | --- | --- |
|  |  |  |  |  |  |  |  |  |
|  |  |  |  |  |  |  |  |  |

−4 −3 −2 −1 0 0.0 0.5 1.0 1.5

####
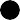

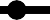
Value

#### Scale


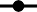
 link


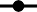
 response

### Gamma groupwise standard deviation

This prior informs the degree to which group-level estimates slope and intercept (trees, species, rounds, dates) are shrunken toward the global mean. With log-link models, it’s important to make this prior fairly tight to keep the model from exploding. Based on my experience with such models, I usually default to a N(0, 1) prior for groupwise standard deviations, but let’s give it some thought.

We need to set a broad enough prior on the sd term to allow species and rounds to vary markedly from one another; I expect a lot less variation across trees and dates. They are all getting the same prior, so the priority is to accommodate the terms for which we expect the most group-level variation.

Suppose the global effect of treatment is -1, meaning that open flowers have on average 37% of the nectar of bagged flowers. We want to allow that some group-level effects achieve near-total depletion, so we want a -4 group-level effect, yielding exp(-5) > 99% depletion. We also want to allow some groups to exceed the mean (of course), perhaps having 0% depletion, which would require a +1 group-level effect. An estimated sd of 2 means that both -4 and (unfortunately) +4 are within the 97.5% interval. That’s probably a bit too wide, but I don’t want to squeeze the low end too much. A N(0, 1) prior will work well here.

For the plot below, the response scale panels show the result of exponentiating the sum of the global treatment effect and 2 x the candidate prior, representing the potential group-level estimates falling with 2*sd of the global mean. The same idea is adapted in the other plot below that depict parameters that get added to other parameters.

**tibble**(N_0_1.0 = **rnorm**(1e5, 0, 1),

N_0_1.5 = **rnorm**(1e5, 0, 1.5),

N_0_2.0 = **rnorm**(1e5, 0, 2), N_0_1.0.res = **exp**(**-**1 **+** 2*****N_0_1.0), N_0_1.5.res = **exp**(**-**1 **+** 2*****N_0_1.5), N_0_2.0.res = **exp**(**-**1 **+** 2*****N_0_2.0)) **%>%**

**pivot_longer**(**everything**(), names_to = "dist") **%>%**

**mutate**(type = **if_else**(**grepl**(".res", dist), "response", "link")) **%>%**

**ggplot**(**aes**(value, fill = type, color = type)) **+**

*#geom_density() +*

**stat_pointinterval**(.width = **c**(0.5, 0.75, .99),

color = "black") **+**

**ylim**(**c**(0, NA)) **+**

**labs**(x = "Value", y = "Probability density", fill = "Scale", color = "Scale") **+ facet_wrap**(**~**dist, scales = "free", ncol = 2) **+**

**scale_y_continuous**(NULL, breaks = NULL)

| **N_0_1.0** | | | | | | | | | | | |
| --- | --- | --- | --- | --- | --- | --- | --- | --- | --- | --- | --- |
|  |  |  |  |  |  |  |  |  |  |  |  |
|  |  |  |  |  |  |  |  |  |  |  |  |

| **N_0_1.0.res** | | | | | | | | |
| --- | --- | --- | --- | --- | --- | --- | --- | --- |
|  |  |  |  |  |  |  |  |  |
|  |  |  |  |  |  |  |  |  |


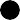

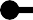
−2 −1 0 1 2 0 20 40 60

| **N_0_1.5** | | | | | | | | | |
| --- | --- | --- | --- | --- | --- | --- | --- | --- | --- |
|  |  |  |  |  |  |  |  |  |  |
|  |  |  |  |  |  |  |  |  |  |


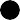


**N_0_1.5.res**


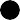
−4 −2 0 2 4 0 250 500 750

| **N_0_2.0** | | | | | | | | | |
| --- | --- | --- | --- | --- | --- | --- | --- | --- | --- |
|  |  |  |  |  |  |  |  |  |  |
|  |  |  |  |  |  |  |  |  |  |

| **N_0_2.0.res** | | | | | | | | | | |
| --- | --- | --- | --- | --- | --- | --- | --- | --- | --- | --- |
|  |  |  |  |  |  |  |  |  |  |  |
|  |  |  |  |  |  |  |  |  |  |  |


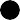

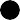
−3 0 3 0 2500 5000 7500 10000

#### Value

#### Scale


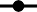
 link


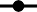
 response

### Binomial global intercept

The global hurdle (hu) parameter is the probability (on the response scale) of getting zeros for treatment

= bagged. This should be relatively low, since we don’t expect a lot of zeros in bagged flowers. They do happen, so we don’t want to make this prior too informative, but a negative bias is definitely justified. N(-2, 2) seems reasonable.

**tibble**(N_n2.0_3.0 = **rnorm**(1e5, **-**2, 3.0),

N_n2.0_2.0 = **rnorm**(1e5, **-**2, 2.0),

N_n1.0_1.5 = **rnorm**(1e5, **-**1, 1.5), N_n2.0_3.0.res = **plogis**(N_n2.0_3.0), N_n2.0_2.0.res = **plogis**(N_n2.0_2.0), N_n1.0_1.5.res = **plogis**(N_n1.0_1.5)) **%>%**

**pivot_longer**(**everything**(), names_to = "dist") **%>%**

**mutate**(type = **if_else**(**grepl**(".res", dist), "response", "link")) **%>%**

**ggplot**(**aes**(value, fill = type, color = type)) **+ geom_density**() **+**

**stat_pointinterval**(.width = **c**(0.5, 0.75, .99),

color = "black") **+**

**ylim**(**c**(0, NA)) **+**

**labs**(x = "Value", y = "Probability density", fill = "Scale", color = "Scale") **+ facet_wrap**(**~**dist, scales = "free", ncol = 2) **+**

**scale_y_continuous**(NULL, breaks = NULL)

| **N_n1.0_1.5.res** | | |
| --- | --- | --- |
|  |  |  |


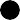


**N_n1.0_1.5**

−7.5 −5.0 −2.5 0.0 2.5 5.0 0.00 0.25 0.50 0.75 1.00

−12 −8 −4 0 4 0.00 0.25 0.50 0.75 1.00


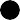


**N_n2.0_2.0**


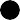


**N_n2.0_2.0.res**


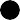


| **N_n2.0_3.0.res** | | |
| --- | --- | --- |
|  |  |  |
|  |  |  |


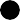


**N_n2.0_3.0**

−10 0 10 0.00 0.25 0.50 0.75 1.00

#### Value

Scale


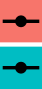
link response


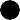


### Binomial global treatment

The treatment hu gets added to the hu intercept on the logit link scale for round == 1. If the hu intercept is

-2 (P(0) = 12%), a treatment hu of +2 would mean that open flowers are 50% likely to have zero nectar in round 1. A treatment hu of +7 would mean that open flowers are empty ~99% of the time. That should be remotely possible. What we should not have is a negative treatment hu, since that would mean open flowers are *less* often empty than bagged ones. N(1.5, 1.25) seems like a good choice.

**tibble**(N_2.0_1.0 = **rnorm**(1e5, 2.0, 1.0),

N_1.5_1.25 = **rnorm**(1e5, 1.5, 1.25),

N_1.0_1.5 = **rnorm**(1e5, 1.0, 1.5),

N_2.0_1.0.res = **plogis**(**-**2 **+** N_2.0_1.0), N_1.5_1.25.res = **plogis**(**-**2 **+** N_1.5_1.25), N_1.0_1.5.res = **plogis**(**-**2 **+** N_1.0_1.5)) **%>%**

**pivot_longer**(**everything**(), names_to = "dist") **%>%**

**mutate**(type = **if_else**(**grepl**(".res", dist), "response", "link")) **%>%**

**ggplot**(**aes**(value, fill = type, color = type)) **+ geom_density**() **+**

**stat_pointinterval**(.width = **c**(0.5, 0.75, .99),

color = "black") **+**

**ylim**(**c**(0, NA)) **+**

**labs**(x = "Value", y = "Probability density", fill = "Scale", color = "Scale") **+ facet_wrap**(**~**dist, scales = "free", ncol = 2) **+**

**scale_y_continuous**(NULL, breaks = NULL)

| **N_1.0_1.5.res** | | |
| --- | --- | --- |
|  |  |  |


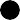


**N_1.0_1.5**

−5 0 5 0.00 0.25 0.50 0.75 1.00

−2.5 0.0 2.5 5.0 0.00 0.25 0.50 0.75 1.00


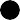


**N_1.5_1.25**


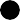


**N_1.5_1.25.res**

#### Scale


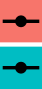
link response


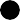

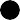


**N_2.0_1.0**


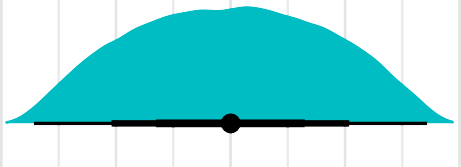


**N_2.0_1.0.res**

−2.5 0.0 2.5 5.0 0.00 0.25 0.50 0.75 1.00

Value

### Binomial groupwise standard deviation

This controls how much the probability of being empty varies across groups. Suppose the hu intercept is 2, meaning very low probability of emptiness in bagged flowers, and the global treatmenthu‘ is 1.5, meaning a mean of 38% emptiness probability. If a group varies by 2, it could be as low as plogis(-0.5 -2)

= 8% empty or as high as plogis(-0.5 + 2) = 82% empty. I want it to be more generous than this, though, so that groups can have nearly 0% or nearly 100% chance of being empty. If we set a prior than makes +/- 5 plausible, that would be comfortably broad enough. N(0, 2) seems reasonable.

**tibble**(N_0_1.0 = **rnorm**(1e5, 0, 1),

N_0_1.5 = **rnorm**(1e5, 0, 1.5),

N_0_2.0 = **rnorm**(1e5, 0, 2),

N_0_1.0.res = **plogis**(**-**2 **+** 1.5 **+** N_0_1.0), N_0_1.5.res = **plogis**(**-**2 **+** 1.5 **+** N_0_1.5), N_0_2.0.5.res = **plogis**(**-**2 **+** 1.5 **+** N_0_2.0)) **%>%**

**pivot_longer**(**everything**(), names_to = "dist") **%>%**

**mutate**(type = **if_else**(**grepl**(".res", dist), "response", "link")) **%>%**

**ggplot**(**aes**(value, fill = type, color = type)) **+ geom_density**() **+**

**stat_pointinterval**(.width = **c**(0.5, 0.75, .99),

color = "black") **+**

**ylim**(**c**(0, NA)) **+**

**labs**(x = "Value", y = "Probability density", fill = "Scale", color = "Scale") **+ facet_wrap**(**~**dist, scales = "free", ncol = 2) **+**

**scale_y_continuous**(NULL, breaks = NULL)


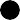


**N_0_1.0**


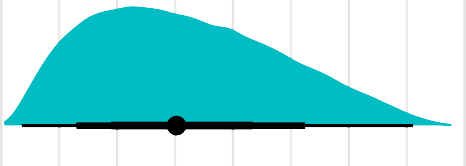


**N_0_1.0.res**

−5.0 −2.5 0.0 2.5 0.00 0.25 0.50 0.75 1.00


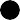


| **N_0_1.5.res** | | |
| --- | --- | --- |
|  |  |  |


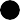


**N_0_1.5**

−4 0 4 0.00 0.25 0.50 0.75 1.00

| **N_0_2.0** | | | | | | | |
| --- | --- | --- | --- | --- | --- | --- | --- |
|  |  |  |  |  |  |  |  |
|  |  |  |  |  |  |  |  |

| **N_0_2.0.5.res** | | |
| --- | --- | --- |
|  |  |  |
|  |  |  |

−5 0 5 0.00 0.25 0.50 0.75 1.00

#### Value


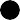

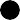


#### Scale


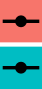
link response

### Call prior set

priors_00 <- **c**(

*# Gamma submodel*

**prior**(**normal**(0, 0.25), class = "Intercept"),

**prior**(**normal**(**-**1, 1),

coef = "treatmentopen"),

**prior**(**normal**(0, 0.5), coef = "round2"),

**prior**(**normal**(0, 0.5), coef = "round3"),

**prior**(**normal**(0, 0.5), coef = "round4"),

**prior**(**normal**(0, 0.5), coef = "round5"),

**prior**(**normal**(**-**1, 1),

coef = "treatmentopen:round2"),

**prior**(**normal**(**-**1, 1),

coef = "treatmentopen:round3"),

**prior**(**normal**(**-**1, 1),

coef = "treatmentopen:round4"),

**prior**(**normal**(**-**1, 1),

coef = "treatmentopen:round5"),

**prior**(**normal**(0, 1),

class = "sd"),

**prior**(**normal**(0, 2.5),

class = shape),

*# Binomial submodel*

**prior**(**normal**(**-**2, 2),

class = "Intercept", dpar = "hu"),

**prior**(**normal**(1.5, 1.25),

coef = "treatmentopen", dpar = "hu"),

**prior**(**normal**(0, 2),

class = "sd", dpar = "hu"),

**prior**(**normal**(0, 1),

coef = "round2", dpar = "hu"),

**prior**(**normal**(0, 1),

coef = "round3", dpar = "hu"),

**prior**(**normal**(0, 1),

coef = "round4", dpar = "hu"),

**prior**(**normal**(0, 1),

coef = "round5", dpar = "hu"),

**prior**(**normal**(1, 1.5),

coef = "treatmentopen:round2", dpar = "hu"),

**prior**(**normal**(1, 1.5),

coef = "treatmentopen:round3", dpar = "hu"),

| *#* | *priors_01* | *<-* | *c(* |
| --- | --- | --- | --- |
| *#* |  |  | *# Gamma submodel* |
| *#* |  |  | *prior(normal(0, 0.25),* |
| *#* |  |  | *class = "Intercept"),* |
| *#* |  |  | *prior(normal(-1, 1),* |
| *#* |  |  | *coef = "treatmentopen"),* |
| *#* |  |  | *prior(normal(0, 1),* |
| *#* |  |  | *class = "sd"),* |
| *#* |  |  | *prior(normal(0, 2.5),* |
| *#* |  |  | *class = "shape"),* |
| *#* |  |  |  |
| *#* |  |  | *# Binomial submodel* |
| *#* |  |  | *prior(normal(-2, 2),* |
| *#* |  |  | *class = "Intercept",* |
| *#* |  |  | *dpar = "hu"),* |
| *#* |  |  | *prior(normal(1.5, 1.25),* |
| *#* |  |  | *coef = "treatmentopen",* |
| *#* |  |  | *dpar = "hu"),* |
| *#* |  |  | *prior(normal(0, 2),* |
| *# class = "sd",*  *# dpar = "hu")*  *# )* | | | |

## Prior-predictive check

**prior**(**normal**(1, 1.5),

coef = "treatmentopen:round4", dpar = "hu"),

**prior**(**normal**(1, 1.5),

coef = "treatmentopen:round5", dpar = "hu")

)

*# brm_00.pp <- brm(bf(nectar.mc ~ treatment*round +*

*# # # # # # # # # #*

*(1 + treatment*round | date) +*

*(1 + treatment*round | species/tree), hu ~ treatment*round +*

*(1 + treatment*round | date) +*

*(1 + treatment*round | species/tree)), family = "hurdle_gamma",*

*prior = priors_00, sample_prior = "only",*

*file = "../output/brm_hubland_00.pp", data = nectar)*

brm_00.pp <- **read_rds**("../output/brm_hubland_00.pp.rds") **pp_check**(brm_00.pp, type = "hist") **+ lims**(x = **c**(0, 20))


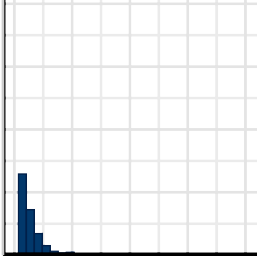

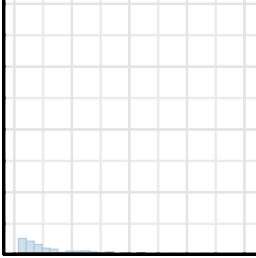

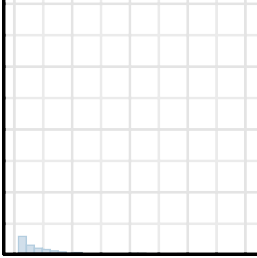

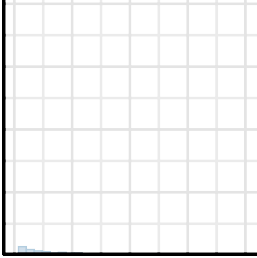


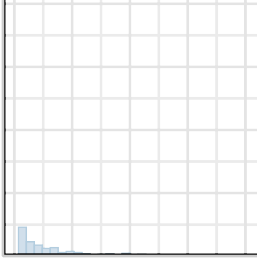

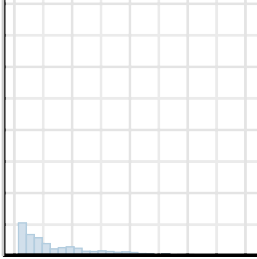

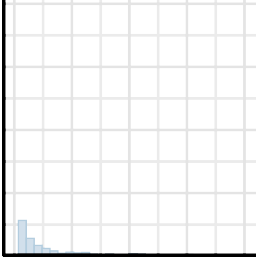

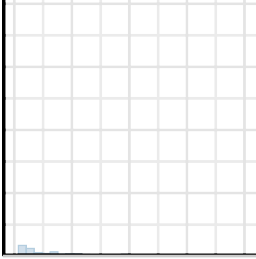

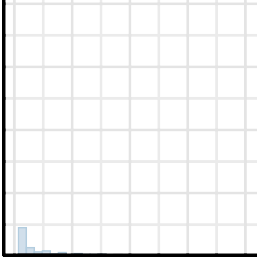

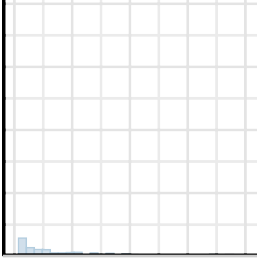

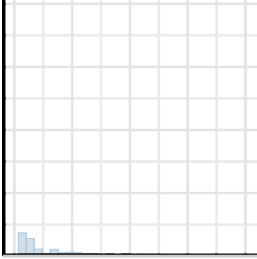

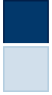
0 5 10 15 20

*y y*rep

0 5 10 15 20 0 5 10 15 20 0 5 10 15 20

## Update model with data

*# brm_00 <- update(brm_00.pp,*

*# file = "../output/brm_hubland_00",*

*# sample_prior = TRUE,*

*# iter = 2000,*

*# control = list(adapt_delta = 0.98))*

brm_00 <- **read_rds**("../output/brm_hubland_00.rds")

**Validate model**

### Posterior predictive check

We want to see that the synthetic data generated by our model are broadly consistent with the real data used to fit it.

**pp_check**(brm_00)

*y y*rep


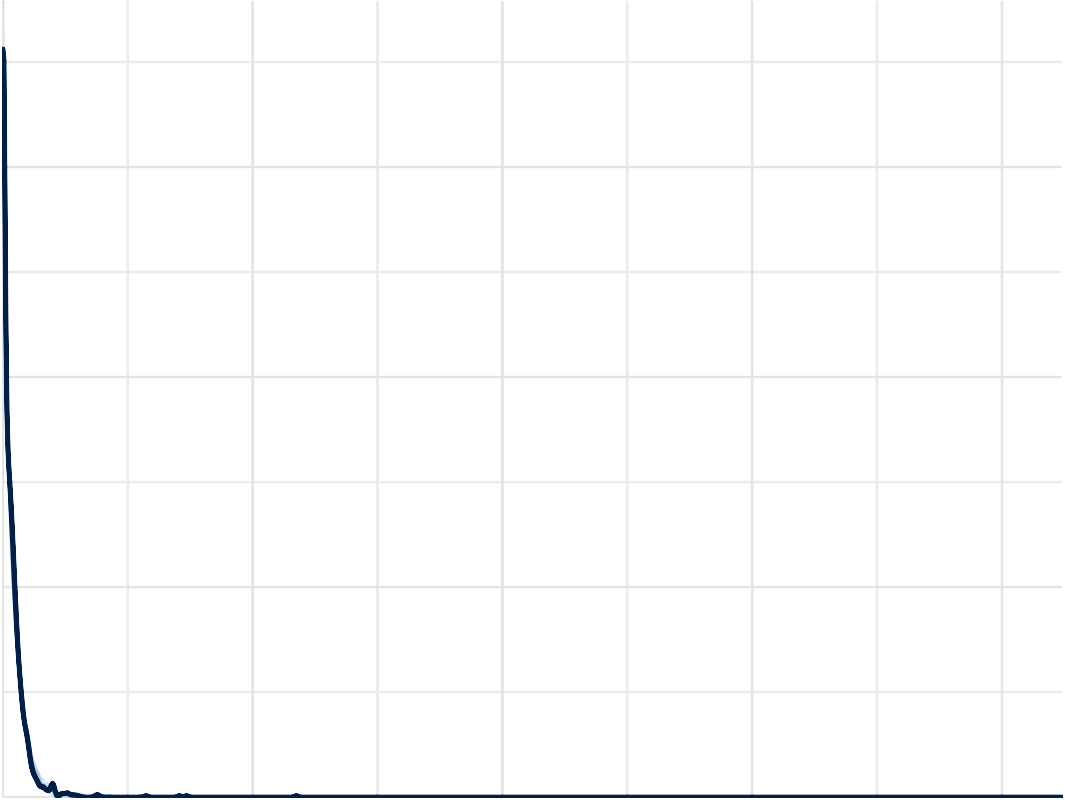


0 25 50 75 100

### Check influence of priors

The goal was for our priors to be weakly informative. What we want to see here is that our priors were reasonably broad relative to our posteriors. If the posterior seems to be fighting the prior, that’s an indication that we misunderstood the prior when we were setting it.

*# Gamma shape*

**mcmc_plot**(brm_00,

variable = **c**("shape", "prior_shape"), regex = FALSE,

type = "areas")

**shape**


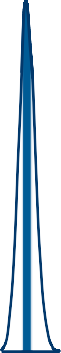


**prior_shape**

0.0 2.5 5.0 7.5

*# Gamma global intercept*

**mcmc_plot**(brm_00,

variable = **c**("b_Intercept", "prior_Intercept"), regex = FALSE,

type = "areas")

**b_Intercept**


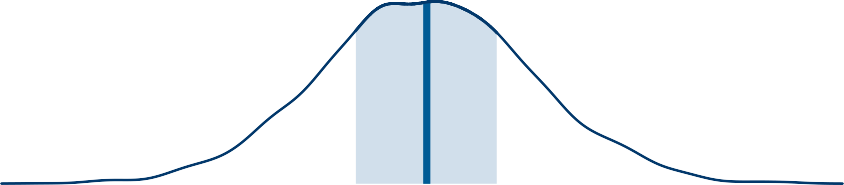


**prior_Intercept**

−1.0 −0.5 0.0 0.5 1.0

*# Gamma global treatment*

**mcmc_plot**(brm_00,

variable = **c**("b_treatmentopen", "prior_b_treatmentopen"), regex = FALSE,

type = "areas")

**b_treatmentopen**


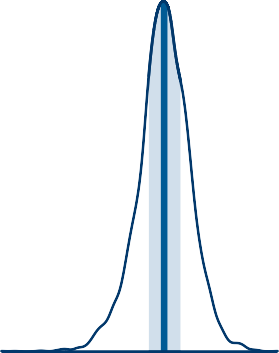


**prior_b_treatmentopen**

−5.0 −2.5 0.0 2.5

*# Gamma global round*

**mcmc_plot**(brm_00,

variable = **c**("b_round2", "prior_b_round2"), regex = FALSE,

type = "areas")

**b_round2**

|  |  |  |  |  |  |  |  |  |  |
| --- | --- | --- | --- | --- | --- | --- | --- | --- | --- |
|  |  |  |  |  |  |  |  |  |  |
|  |  |  |  |  |  |  |  |  |  |

**prior_b_round2**


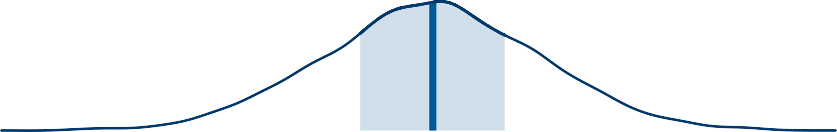


−2 −1 0 1 2


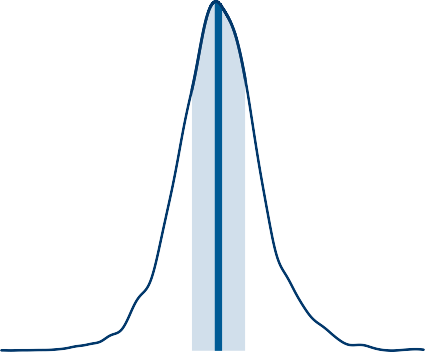


**mcmc_plot**(brm_00,

variable = **c**("b_round3", "prior_b_round3"), regex = FALSE,

type = "areas")

**b_round3**

|  |  |  |  |  |  |  |  |  |  |
| --- | --- | --- | --- | --- | --- | --- | --- | --- | --- |
|  |  |  |  |  |  |  |  |  |  |
|  |  |  |  |  |  |  |  |  |  |

**prior_b_round3**


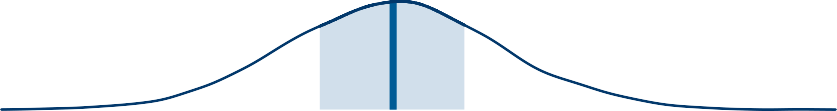


−2 −1 0 1 2


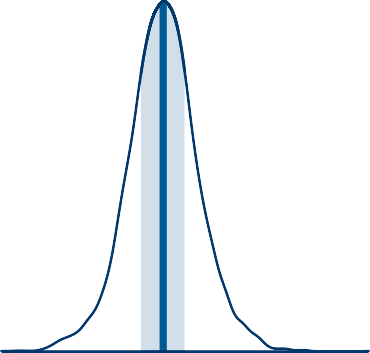


**mcmc_plot**(brm_00,

variable = **c**("b_round4", "prior_b_round4"), regex = FALSE,

type = "areas")

**b_round4**

|  |  |  |  |  |  |  |  |  |  |
| --- | --- | --- | --- | --- | --- | --- | --- | --- | --- |
|  |  |  |  |  |  |  |  |  |  |
|  |  |  |  |  |  |  |  |  |  |

**prior_b_round4**


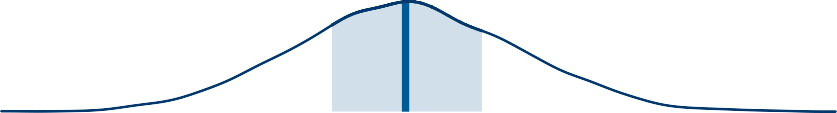


−2 −1 0 1 2


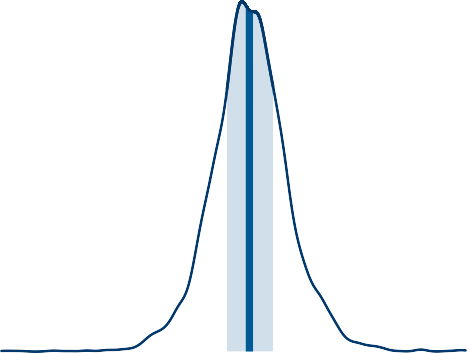


**mcmc_plot**(brm_00,

variable = **c**("b_round5", "prior_b_round5"), regex = FALSE,

type = "areas")

**b_round5**


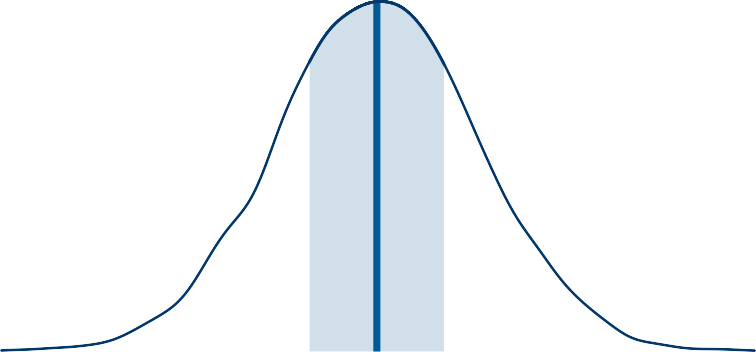


**prior_b_round5**

−2 −1 0 1 2

*# Gamma treatment * round*

**mcmc_plot**(brm_00,

variable = **c**("b_treatmentopen:round2", "prior_b_treatmentopen:round2"), regex = FALSE,

type = "areas")

**b_treatmentopen:round2**

|  |  |  |  |  |  |  |  |  |  |
| --- | --- | --- | --- | --- | --- | --- | --- | --- | --- |
|  |  |  |  |  |  |  |  |  |  |
|  |  |  |  |  |  |  |  |  |  |

**prior_b_treatmentopen:round2**


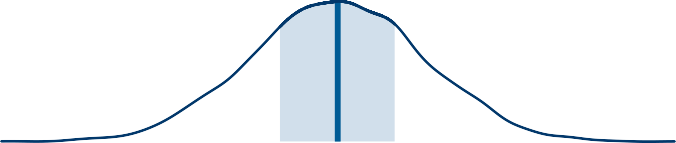


−4 −2 0 2


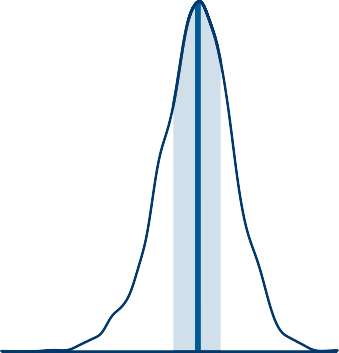


**mcmc_plot**(brm_00,

variable = **c**("b_treatmentopen:round3", "prior_b_treatmentopen:round3"), regex = FALSE,

type = "areas")

**b_treatmentopen:round3**

|  |  |  |  |  |  |  |  |
| --- | --- | --- | --- | --- | --- | --- | --- |
|  |  |  |  |  |  |  |  |
|  |  |  |  |  |  |  |  |

**prior_b_treatmentopen:round3**


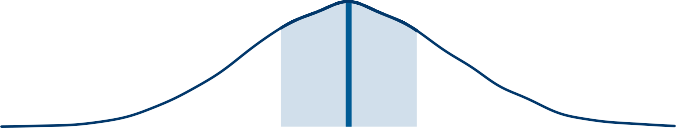


−5.0 −2.5 0.0 2.5


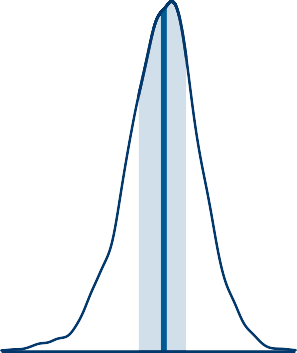


**mcmc_plot**(brm_00,

variable = **c**("b_treatmentopen:round4", "prior_b_treatmentopen:round4"), regex = FALSE,

type = "areas")


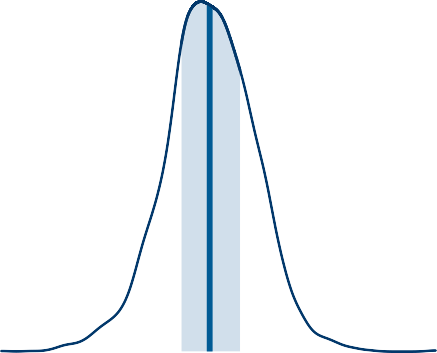
**b_treatmentopen:round4**

|  |  |  |  |  |  |  |  |
| --- | --- | --- | --- | --- | --- | --- | --- |
|  |  |  |  |  |  |  |  |
|  |  |  |  |  |  |  |  |

**prior_b_treatmentopen:round4**


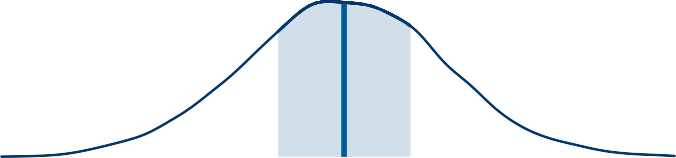


−5.0 −2.5 0.0 2.5

**mcmc_plot**(brm_00,

variable = **c**("b_treatmentopen:round5", "prior_b_treatmentopen:round5"), regex = FALSE,

type = "areas")


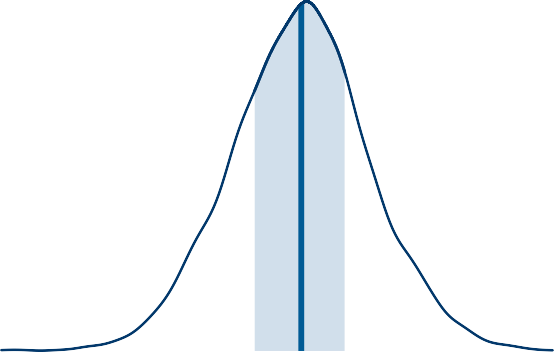
**b_treatmentopen:round5**

|  |  |  |  |  |  |  |  |
| --- | --- | --- | --- | --- | --- | --- | --- |
|  |  |  |  |  |  |  |  |
|  |  |  |  |  |  |  |  |

**prior_b_treatmentopen:round5**


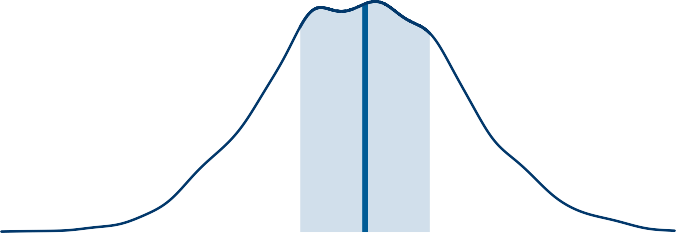


−5.0 −2.5 0.0 2.5

*# Binomial global intercept*

**mcmc_plot**(brm_00,

variable = **c**("b_hu_Intercept", "prior_Intercept_hu"), regex = FALSE,

type = "areas")


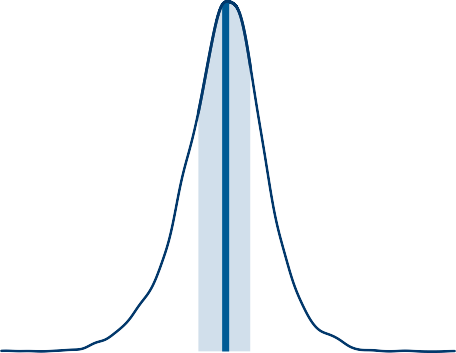
**b_hu_Intercept**

|  |  |  |  |  |  |  |
| --- | --- | --- | --- | --- | --- | --- |
|  |  |  |  |  |  |  |
|  |  |  |  |  |  |  |


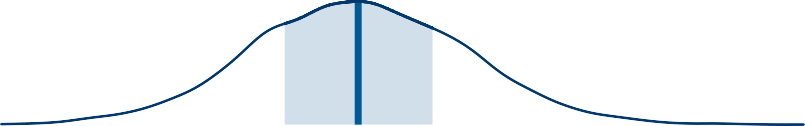
**prior_Intercept_hu**

−10 −5 0 5

*# Binomial global treatment*

**mcmc_plot**(brm_00,

variable = **c**("b_hu_treatmentopen", "prior_b_hu_treatmentopen"), regex = FALSE,

type = "areas")


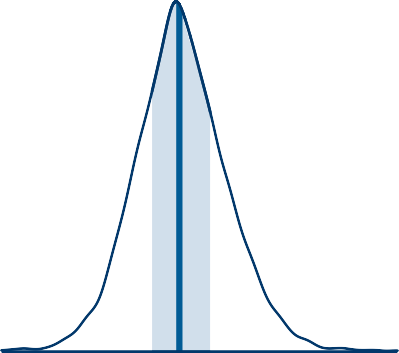
**b_hu_treatmentopen**

|  |  |  |  |  |  |  |  |  |  |  |
| --- | --- | --- | --- | --- | --- | --- | --- | --- | --- | --- |
|  |  |  |  |  |  |  |  |  |  |  |
|  |  |  |  |  |  |  |  |  |  |  |

**prior_b_hu_treatmentopen**


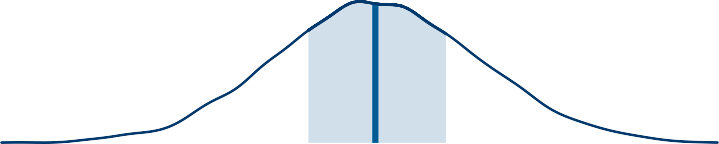


−2 0 2 4 6

*# Binomial global round*

**mcmc_plot**(brm_00,

variable = **c**("b_hu_round2", "prior_b_hu_round2"), regex = FALSE,

type = "areas")

**b_hu_round2**

|  |  |  |  |  |  |  |  |
| --- | --- | --- | --- | --- | --- | --- | --- |
|  |  |  |  |  |  |  |  |
|  |  |  |  |  |  |  |  |

**prior_b_hu_round2**


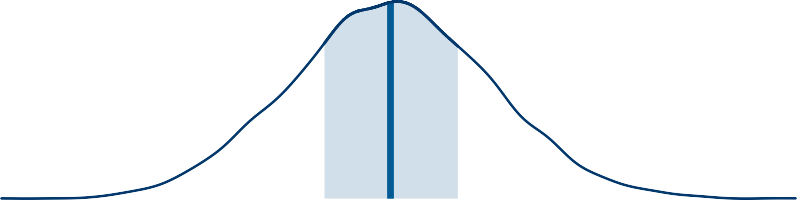


−2.5 0.0 2.5


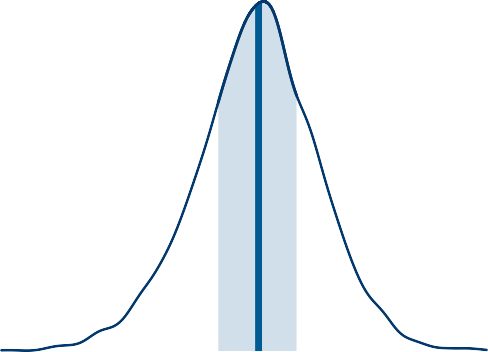


**mcmc_plot**(brm_00,

variable = **c**("b_hu_round3", "prior_b_hu_round3"), regex = FALSE,

type = "areas")


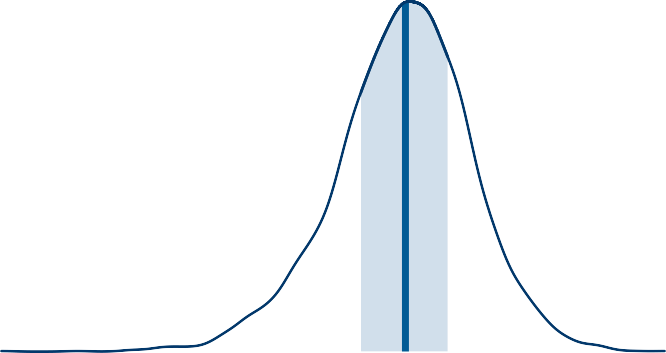
**b_hu_round3**

|  |  |  |  |  |  |  |  |  |
| --- | --- | --- | --- | --- | --- | --- | --- | --- |
|  |  |  |  |  |  |  |  |  |
|  |  |  |  |  |  |  |  |  |

**prior_b_hu_round3**


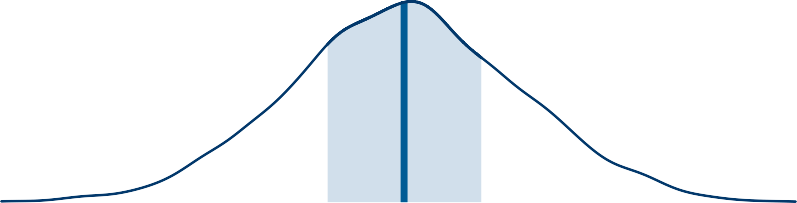


−4 −2 0 2 4

**mcmc_plot**(brm_00,

variable = **c**("b_hu_round4", "prior_b_hu_round4"), regex = FALSE,

type = "areas")


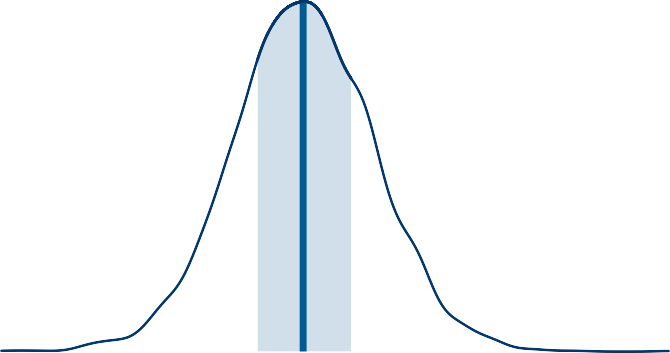
**b_hu_round4**

|  |  |  |  |  |  |  |  |
| --- | --- | --- | --- | --- | --- | --- | --- |
|  |  |  |  |  |  |  |  |
|  |  |  |  |  |  |  |  |

**prior_b_hu_round4**


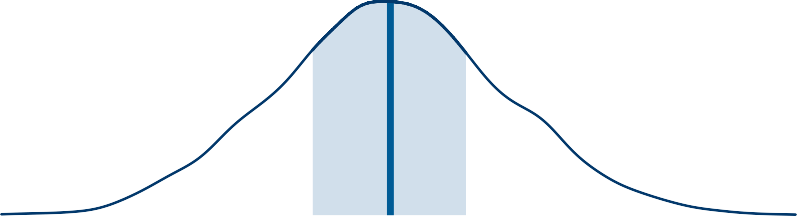


−2 0 2 4

**mcmc_plot**(brm_00,

variable = **c**("b_hu_round5", "prior_b_hu_round5"), regex = FALSE,

type = "areas")


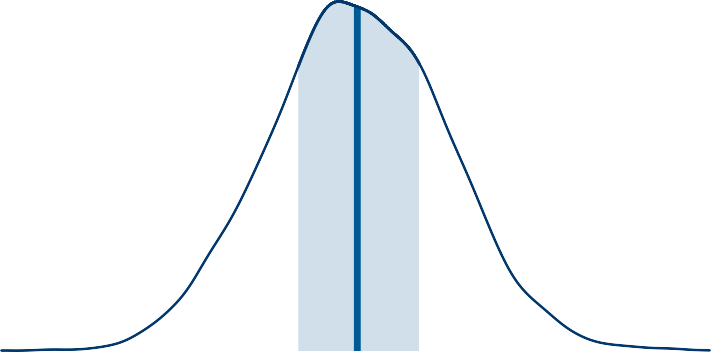
**b_hu_round5**

|  |  |  |  |  |  |  |  |  |
| --- | --- | --- | --- | --- | --- | --- | --- | --- |
|  |  |  |  |  |  |  |  |  |
|  |  |  |  |  |  |  |  |  |

**prior_b_hu_round5**


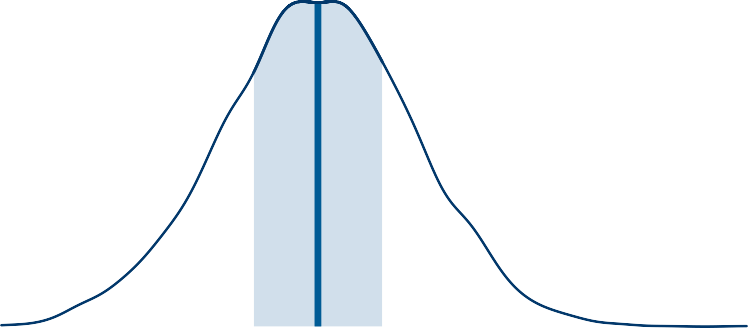


−2.5 0.0 2.5 5.0

*# Binomial treatment * round*

**mcmc_plot**(brm_00,

variable = **c**("b_hu_treatmentopen:round2", "prior_b_hu_treatmentopen:round2"), regex = FALSE,

type = "areas")


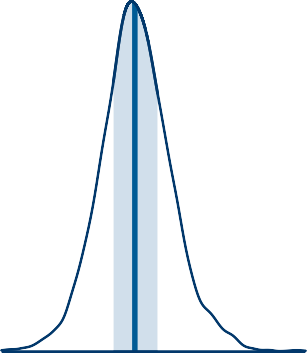
**b_hu_treatmentopen:round2**

|  |  |  |  |  |  |  |  |  |  |  |
| --- | --- | --- | --- | --- | --- | --- | --- | --- | --- | --- |
|  |  |  |  |  |  |  |  |  |  |  |
|  |  |  |  |  |  |  |  |  |  |  |

**prior_b_hu_treatmentopen:round2**


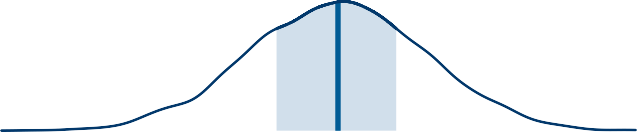


−5.0 −2.5 0.0 2.5 5.0

**mcmc_plot**(brm_00,

variable = **c**("b_hu_treatmentopen:round3", "prior_b_hu_treatmentopen:round3"), regex = FALSE,

type = "areas")


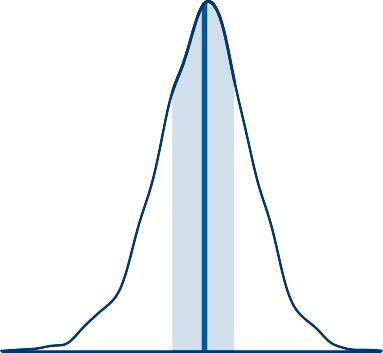
**b_hu_treatmentopen:round3**

|  |  |  |  |  |  |  |  |  |  |
| --- | --- | --- | --- | --- | --- | --- | --- | --- | --- |
|  |  |  |  |  |  |  |  |  |  |
|  |  |  |  |  |  |  |  |  |  |

**prior_b_hu_treatmentopen:round3**


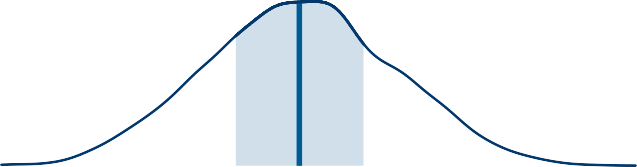


−2.5 0.0 2.5 5.0

**mcmc_plot**(brm_00,

variable = **c**("b_hu_treatmentopen:round4", "prior_b_hu_treatmentopen:round4"), regex = FALSE,

type = "areas")


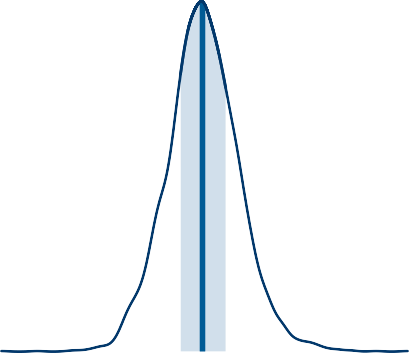
**b_hu_treatmentopen:round4**

|  |  |  |  |  |  |  |  |  |  |  |
| --- | --- | --- | --- | --- | --- | --- | --- | --- | --- | --- |
|  |  |  |  |  |  |  |  |  |  |  |
|  |  |  |  |  |  |  |  |  |  |  |

**prior_b_hu_treatmentopen:round4**


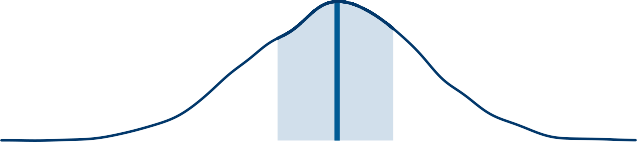


−5.0 −2.5 0.0 2.5 5.0 7.5

**mcmc_plot**(brm_00,

variable = **c**("b_hu_treatmentopen:round5", "prior_b_hu_treatmentopen:round5"), regex = FALSE,

type = "areas")


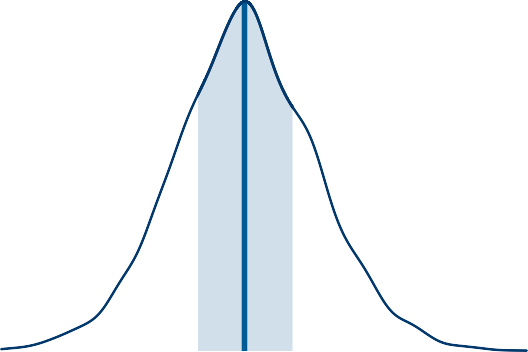
**b_hu_treatmentopen:round5**

|  |  |  |  |  |  |  |  |  |  |  |
| --- | --- | --- | --- | --- | --- | --- | --- | --- | --- | --- |
|  |  |  |  |  |  |  |  |  |  |  |
|  |  |  |  |  |  |  |  |  |  |  |

**prior_b_hu_treatmentopen:round5**


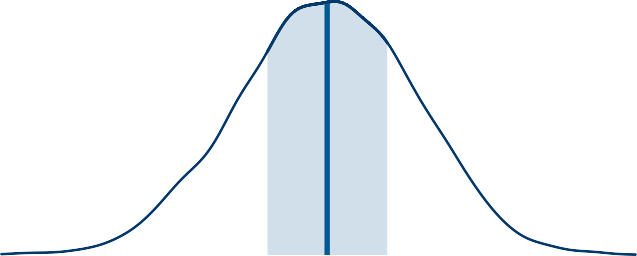


−5.0 −2.5 0.0 2.5 5.0

*# Gamma sd parameters*

**mcmc_plot**(brm_00,

variable = **c**("sd_date Intercept",

"sd_date treatmentopen", "sd_species Intercept", "sd_species treatmentopen", "prior_sd_date", "prior_sd_species"),

regex = FALSE, type = "areas")

**sd_date Intercept**


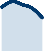

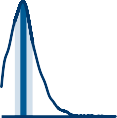

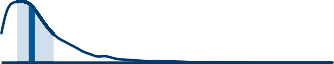

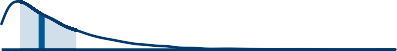


**sd_date treatmentopen**

**sd_species Intercept**

**sd_species treatmentopen**

**prior_sd_date**

**prior_sd_species**

0 1 2 3 4

*# Binomial sd parameters*

**mcmc_plot**(brm_00,

variable = **c**("sd_date hu_Intercept",

"sd_date hu_treatmentopen", "sd_species hu_Intercept", "sd_species hu_treatmentopen", "prior_sd_species 1"),

regex = FALSE, type = "areas")

**sd_date hu_Intercept**


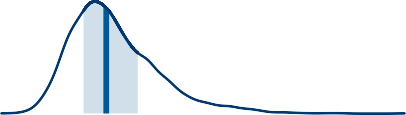


**sd_date hu_treatmentopen**

**sd_species hu_Intercept**

**sd_species hu_treatmentopen**

**prior_sd_species 1**

0 2 4 6 8

Gabry, Jonah, Daniel Simpson, Aki Vehtari, Michael Betancourt, and Andrew Gelman. 2019. “Visualization in Bayesian Workflow.” *Journal of the Royal Statistical Society. Series A,* 182 (2): 389–402.

Gelman, Andrew. 2005. “Analysis of Variance: Why It Is More Important Than Ever.” *Annals of Statistics*

33 (1): 1–31.

Wesner, Jeff S, and Justin P F Pomeranz. 2021. “Choosing Priors in Bayesian Ecological Models by Simulating from the Prior Predictive Distribution.” *Ecosphere* 12 (9).

# Pollinator competition and the contingency of nectar depletion during an early spring resource pulse

Appendix 2: Model processing and visualization

Douglas B. Sponsler* Murray Hamilton Michael Wiesneth Ingolf Steffan-Dewenter

2024-05-23

| **Contents**  [**Summary**](file:///\\172.24.191.130\Straive_E3\Journals\WILEY_JNLS\RAW\ECE3\ECE3_11531\FPP\doc\ECE3_11531_AM.docx#_bookmark16) | **1** |
| --- | --- |
| [**Packages**](file:///\\172.24.191.130\Straive_E3\Journals\WILEY_JNLS\RAW\ECE3\ECE3_11531\FPP\doc\ECE3_11531_AM.docx#_bookmark17) | **3** |
| [**Load data and models**](file:///\\172.24.191.130\Straive_E3\Journals\WILEY_JNLS\RAW\ECE3\ECE3_11531\FPP\doc\ECE3_11531_AM.docx#_bookmark18) | **3** |
| [**Study area (Figure S1)**](file:///\\172.24.191.130\Straive_E3\Journals\WILEY_JNLS\RAW\ECE3\ECE3_11531\FPP\doc\ECE3_11531_AM.docx#_bookmark19) | **3** |
| [**Sampling methods (Figure 1)**](file:///\\172.24.191.130\Straive_E3\Journals\WILEY_JNLS\RAW\ECE3\ECE3_11531\FPP\doc\ECE3_11531_AM.docx#_bookmark20) | **4** |
| [**Nectary morphology of sampled species (Figure S2)**](file:///\\172.24.191.130\Straive_E3\Journals\WILEY_JNLS\RAW\ECE3\ECE3_11531\FPP\doc\ECE3_11531_AM.docx#_bookmark21) | **5** |
| [**Sampling patterns (Figure S3)**](file:///\\172.24.191.130\Straive_E3\Journals\WILEY_JNLS\RAW\ECE3\ECE3_11531\FPP\doc\ECE3_11531_AM.docx#_bookmark22) | **5** |
| [**Generate posterior predictions from model**](file:///\\172.24.191.130\Straive_E3\Journals\WILEY_JNLS\RAW\ECE3\ECE3_11531\FPP\doc\ECE3_11531_AM.docx#_bookmark23) | **7** |
| [**Average depletion rate by species x round x day (Figure 2)**](file:///\\172.24.191.130\Straive_E3\Journals\WILEY_JNLS\RAW\ECE3\ECE3_11531\FPP\doc\ECE3_11531_AM.docx#_bookmark24) | **8** |
| [**Weather as constraint on pollinator activity (Figure S4)**](file:///\\172.24.191.130\Straive_E3\Journals\WILEY_JNLS\RAW\ECE3\ECE3_11531\FPP\doc\ECE3_11531_AM.docx#_bookmark25) | **9** |
| [**Dew dilutes nectar**](file:///\\172.24.191.130\Straive_E3\Journals\WILEY_JNLS\RAW\ECE3\ECE3_11531\FPP\doc\ECE3_11531_AM.docx#_bookmark26) | **11** |
| [**Average depletion rate by species x date x tree (Figure S5)**](file:///\\172.24.191.130\Straive_E3\Journals\WILEY_JNLS\RAW\ECE3\ECE3_11531\FPP\doc\ECE3_11531_AM.docx#_bookmark27) | **13** |
| **Summary**  We begin by generating predicted nectar volumes based on the posterior distributions of our models. | By |

using tidybayes::add_epred_draws(), which is a wrapper for brms::posterior_epred(), we are getting the expected *mean* of normalized nectar volume for bagged and open flowers. This is the biologically relevant metric because it is the mean reward that relates most directly to the fitness of a forager.

We then marginalize these predictions to get the distribution of average expected nectar volume across treatment, species, tree, round, and date. There are various ways to calculate marginal and/or conditional predictions. Our approach is to calculate the so-called “group average marginal prediction” by grouping predictions by one or more focal variables and averaging over the others. See this excellent post by Andrew Heiss for details:

<https://www.andrewheiss.com/blog/2022/05/20/marginalia/>

We then calculate depletion by plugging these group average marginal predictions into the equation below:

*Vopen*

= 1

*D −*

*Vbagged*

(1)

## Packages

**Load data and models**

nectar <- **read_rds**("../data/processed/hubland_nectar.rds") pollen_bees <- **read_rds**("../data/processed/hubland_pollenbees.rds")

concentrations <- **read_rds**("../data/processed/hubland_concentrations.rds") weather <- **read_rds**("../data/processed/hubland_weather.rds")

brm_hubland_00 <- **read_rds**("../output/brm_hubland_00.rds")

**Study area (Figure S1)**

Figure 1: Map of study area. Note that the two blackthorn locations indicated on the map each represent a linear hedge including many individual shrubs, of which we sampled a total of 27. SatellIte imagery was provided by Google Earth, accessed via QGIS. Note that this image was taken in the early spring of 2022, exactly a year before our study, and flowering plum trees are visible as white patches within the forested areas.

## Sampling methods (Figure 1)

Illustration of sampling methods. Trees selected for sampling were bagged early in the morning, before the onset of pollinator activity (A). Bags were constructed of a fine synthetic mesh that effectively excluded insect visitors while allowing air to flow relatively unobstructed (B). Nectar was sampled by probing each flower with a microcapillary tube (C); in this image, a small volume of nectar is visible below the meniscus

near the distal end of the tube.

## Nectary morphology of sampled species (Figure S2)

Cross-sectional illustrations of nectary geometry (dark green area) in *Prunus domestica*, *Prunus spinosa*, *Prunus* (Cerasus), and *Pyrus communis*. The illustration of *Prunus spinosa* is a vectorization of a public domain image from plantillustrations.org, originally from A. Masclef, *Atlas des plantes de France* (1890-1893). Illustrations of the other three species are based on photographs taken by the authors during the course of this study.

## Sampling patterns (Figure S3)

### Sampling summary

flower_count <- nectar **%>% group_by**(treatment) **%>% summarise**(n = **n**())

tree_count <- nectar **%>% select**(species, tree) **%>% group_by**(species) **%>%**

**summarize**(trees = **length**(**unique**(tree)))

flower_count

## # A tibble: 2 x 2

## treatment n ## <chr> <int>

## 1 bagged 621

## 2 open 627

tree_count

## # A tibble: 4 x 2

## species trees

## <chr> <int> ## 1 Prunus_cherry 4

## 2 Prunus_domestica 12

## 3 Prunus_spinosa 27

## 4 Pyrus 4

### Sampling timeline

samples <- nectar **%>%**

**mutate**(flower = **row_number**()) **%>% select**(flower, date, species, tree) **%>% distinct**() **%>%**

**mutate**(species = **case_when**(

species **==** "Prunus_cherry" **~** "Prunus (Cerasus)", species **==** "Prunus_domestica" **~** "Prunus domestica", species **==** "Prunus_spinosa" **~** "Prunus spinosa", species **==** "Pyrus" **~** "Pyrus communis"

))

**ggplot**(samples, **aes**(date, fill = species)) **+ geom_bar**() **+**

**labs**(x = NULL, y = "Flowers sampled", fill = "Species") **+ theme_lucid**()

300

200

Flowers sampled

100

#### Species

Prunus (Cerasus) Prunus domestica Prunus spinosa

Pyrus communis

0

Mar 20 Mar 27 Apr 03 Apr 10 Apr 17

*# ggsave("../writing/plum_paper/figures/figS3.pdf", width = 7, height = 3) # ggsave("../writing/plum_paper/figures/figS3.png", width = 7, height = 3)*

## Generate posterior predictions from model

By using tidybayes::add_epred_draws(), which is a wrapper for brms::posterior_epred(), we are getting the expected *mean* nectar volume. This is the biologically relevant metric because it is the mean reward that relates most directly to the fitness of a forager.

*# New data frame to generate predictions for the full design matrix of the model*

nd <- nectar **%>%**

**select**(treatment, round, date, species, tree) **%>% distinct**()

*# Predictions from full design matrix*

brm_hubland_00_epred <- brm_hubland_00 **%>% add_epred_draws**(

newdata = nd, re_formula = NULL,

allow_new_levels = FALSE, dpar = TRUE)

## Average depletion rate by species x round x day (Figure 2)

### Calculate conditional means

brm_hubland_00_epred.round_species_day <- brm_hubland_00_epred **%>% group_by**(treatment, round, species, date, .draw) **%>% summarize**(ame = **mean**(.epred))

## `summarise()` has grouped output by 'treatment', 'round', 'species', 'date'. ## You can override using the `.groups` argument.

**Calculate mean (censored) depletion**

depletion.round_species_day <- brm_hubland_00_epred.round_species_day **%>% pivot_wider**(names_from = treatment, values_from = ame) **%>% mutate**(depletion = 1 **-** (open**/**bagged),

depletion.c = **if_else**(depletion **>** 0, depletion, 0)) **%>% mutate**(common.name = **case_when**(

species **==** "Prunus_cherry" **~** "cherry", species **==** "Prunus_domestica" **~** "plum", species **==** "Prunus_spinosa" **~** "blackthorn", species **==** "Pyrus" **~** "pear"

)) **%>%**

**mutate**(time = **case_when**( round **==** 1 **~** "09:00",

round **==** 2 **~** "11:00",

round **==** 3 **~** "13:00",

round **==** 4 **~** "15:00",

round **==** 5 **~** "17:00"

)) **%>%**

**mutate**(time = **parse_time**(time))

**Summarize**

depletion.round_species_day.sum <- depletion.round_species_day **%>% group_by**(common.name, round, date) **%>% point_interval**(depletion.c)

**Visualize**

**ggplot**(depletion.round_species_day,

**aes**(x = **hour**(time), y = depletion.c*****100, color = common.name)) **+ stat_pointinterval**(point_interval = "median_hdci", position = **position_dodge**(width = 0.8)) **+ labs**(x = "Hour", y = "Depletion (%)", color = "Species") **+**

**scale_x_continuous**(breaks = **c**(9, 11, 13, 15, 17),

labels = **c**("9:00", "11:00", "13:00", "15:00", "17:00")) **+**

**scale_color_manual**(values = **c**("#00BFC4", "#F8766D", "#7CAE00", "#C77CFF")) **+ facet_wrap**(**~**date) **+**

**theme_light**() **+**

**theme**(panel.grid.minor = **element_blank**(),

axis.text.x = **element_text**(angle = 45, vjust = 1, hjust=1))

100

2023−03−20

75

50

25

0

2023−03−29

100

Depletion (%)

75

50

25

0

#### Species

2023−03−21

2023−03−22

blackthorn cherry pear

2023−04−10

plum

100

2023−04−14

2023−04−06

2023−04−19

2023−04−21

75

50

25

0

9:00

11:00

13:00

15:00

17:00

9:00

11:00

13:00

15:00

17:00

9:00

11:00

13:00

15:00

17:00

Hour

*# ggsave("../writing/plum_paper/figures/fig2.pdf", width= 9, height = 7) # ggsave("../writing/plum_paper/figures/fig2.png", width= 9, height = 7)*

## Weather as constraint on pollinator activity (Figure S4)

In panel A, each point represents an hourly mean of weather data during daylight hours. Blue points are those for which temperature was at least 10C and during which no precipitation occurred, conditions suitable for pollinator foraging. Our sampling activity is depicted in the pink rugplot. Panel B shows the daily proportion of daylight hours that met the criteria of foraging conditions, and the horizontal blue line indicates the mean proportion across all days.

weather_foraging <- weather **%>% filter**(sun.wh **>** 0) **%>% group_by**(foraging) **%>% summarize**(hours = **n**()) **%>% mutate**(hours.prop = hours**/sum**(hours))

weather_foraging_daily <- weather **%>% filter**(sun.wh **>** 0) **%>%**

**select**(date, time, foraging) **%>% group_by**(date) **%>% mutate**(day.hours = **n**()) **%>%**

**group_by**(date, foraging, day.hours) **%>% summarize**(hours = **n**()) **%>% mutate**(foraging.hours = foraging*****hours) **%>% group_by**(date, day.hours) **%>%**

**summarize**(foraging.hours = **sum**(foraging.hours)) **%>% mutate**(prop.foraging.hours = foraging.hours**/**day.hours)

## `summarise()` has grouped output by 'date', 'foraging'. You can override using ## the `.groups` argument.

## `summarise()` has grouped output by 'date'. You can override using the ## `.groups` argument.

weather_long <- weather **%>% filter**(sun.wh **>** 0) **%>%**

**pivot_longer**(cols = **c**(temp.mean, precip.mm, sun.wh), values_to = "value",

names_to = "variable") **%>% mutate**(foraging = **factor**(foraging)) **%>% mutate**(datetime = **as_datetime**(**paste**(date, time)))

weather_p1 <- **ggplot**(weather_foraging_daily, **aes**(date, prop.foraging.hours)) **+ geom_point**() **+**

**geom_line**() **+**

**geom_smooth**(method = "lm", formula = y **~** 1, color = "dodgerblue", se = FALSE) **+ labs**(y = "Proportion of daylight hours", x = "Date") **+**

**theme_lucid**()

weather_p2 <- **ggplot**(**filter**(weather_long, variable **==** "temp.mean"),

**aes**(datetime, value)) **+ geom_line**(color = "gray60") **+ geom_point**(**aes**(color = foraging)) **+**

**geom_hline**(yintercept = 10, linetype = "dashed") **+ geom_rug**(data = nectar, **aes**(datetime),

color = "hotpink", length = **unit**(0.05, "npc"), inherit.aes = FALSE) **+ labs**(x = NULL, y = "Mean temperature (C)", color = "Foraging") **+ scale_color_manual**(values = **c**("gray40", "dodgerblue"),

labels = **c**(FALSE, TRUE)) **+**

**theme_lucid**() **+**

**theme**(axis.title.x = **element_blank**(), axis.text.x = **element_blank**())

weather_p <- (weather_p2 **/** weather_p1) **+ plot_annotation**(tag_levels = "A") weather_p

#### A

Mean temperature (C)

20

15

10

5

0

#### B

Proportion of daylight hours

1.00

0.75

0.50

0.25

0.00

Mar 15 Apr 01 Apr 15

#### Date

#### Foraging

FALSE TRUE

*# ggsave("../writing/plum_paper/figures/fig3.pdf", weather_p, height = 7, width = 8.5) # ggsave("../writing/plum_paper/figures/fig3.png", weather_p, height = 7, width = 8.5)*

## Dew dilutes nectar

dew <- nectar **%>%**

**filter**(date **==** "2023-04-21")

concentrations_dew <- concentrations **%>% inner_join**(dew) **%>%**

**group_by**(date, time, round, hour, tree, treatment, concentration) **%>% summarize**(mean.nectar.ul = **mean**(nectar.ul))

## Joining with `by = join_by(date, time, round, hour, tree, treatment)`

## `summarise()` has grouped output by 'date', 'time', 'round', 'hour', 'tree', ## 'treatment'. You can override using the `.groups` argument.

dew_p1 <- **ggplot**(concentrations_dew, **aes**(hour, concentration, fill = treatment)) **+ geom_point**(position = **position_dodge**(width = 0.2), alpha = 0.75, pch = 21, color = "black") **+ labs**(x = NULL, y = "Concentration (%)",

fill = "Treatment") **+ scale_fill_grey**() **+**

**theme_lucid**(tags.size = 8, axis.text.size = 8, legend.text.size = 8,

legend.title.size = 8, axis.title.size = 8, axis.title.space = 5) **+**

**theme_lucid**(tags.size = 10, axis.text.size = 10, legend.text.size = 10,

legend.title.size = 10, axis.title.size = 10, axis.title.space = 5) **+**

**theme**(axis.text.x = **element_blank**()) **+ theme**(panel.grid.major = **element_blank**(),

panel.grid.minor = **element_blank**())

dew_p2 <- **ggplot**(dew, **aes**(hour, nectar.ul, fill = treatment)) **+ geom_violin**(scale = "width", alpha = 0.5, color = "black") **+ scale_fill_grey**() **+**

**labs**(x = NULL, y = "Volume (uL)",

color = "Treatment", fill = "Treatment") **+**

**theme_lucid**(tags.size = 10, axis.text.size = 10, legend.text.size = 10,

legend.title.size = 10, axis.title.size = 10, axis.title.space = 5) **+**

**theme**(panel.grid.major = **element_blank**(), panel.grid.minor = **element_blank**())

dew_flower2 <- **image_read**("../writing/plum_paper/figures/dew.jpg") **%>% image_ggplot**() **+**

**theme_lucid**(tags.size = 10, axis.text.size = 10, legend.text.size = 10,

legend.title.size = 10, axis.title.size = 10, axis.title.space = 5) **+**

**theme**(axis.title.x = **element_blank**(), axis.title.y = **element_blank**(), axis.text.x = **element_blank**(), axis.text.y = **element_blank**(), axis.ticks.x = **element_blank**(), axis.ticks.y = **element_blank**(), axis.line.x = **element_blank**(), axis.line.y = **element_blank**())

dew_p <- (dew_flower2 **|** (dew_p1 **/** dew_p2)) **+ plot_layout**(guides = 'collect') **+ plot_annotation**(tag_levels = "A")

dew_p

## Warning: Removed 5 rows containing missing values (`geom_point()`).

A B

Concentration (%)

30

20

10

0

C

15

Volume (uL)

10

5

0

09:00 11:00 13:00 15:00

Treatment

bagged open

Treatment

bagged open

*# ggsave("../writing/plum_paper/figures/fig3.pdf", dew_p, height = 3.5, width = 6.5) # ggsave("../writing/plum_paper/figures/fig3.png", dew_p, height = 3.5, width = 6.5)*

## Average depletion rate by species x date x tree (Figure S5)

### Calculate conditional means

brm_hubland_00_epred.species_day_tree <- brm_hubland_00_epred **%>% group_by**(treatment, species, date, tree, .draw) **%>% summarize**(ame = **mean**(.epred))

## `summarise()` has grouped output by 'treatment', 'species', 'date', 'tree'. You ## can override using the `.groups` argument.

**Calculate depletion**

depletion.species_day_tree <- brm_hubland_00_epred.species_day_tree **%>% pivot_wider**(names_from = treatment, values_from = ame) **%>% mutate**(depletion = 1 **-** (open**/**bagged),

depletion.c = **if_else**(depletion **>** 0, depletion, 0)) **%>% mutate**(latin.name = **case_when**(

species **==** "Prunus_cherry" **~** "Prunus (Cerasus)", species **==** "Prunus_domestica" **~** "Prunus domestica", species **==** "Prunus_spinosa" **~** "Prunus spinosa", species **==** "Pyrus" **~** "Pyrus communis"

)) **%>%**

**mutate**(common.name = **case_when**(

species **==** "Prunus_cherry" **~** "cherry", species **==** "Prunus_domestica" **~** "plum", species **==** "Prunus_spinosa" **~** "blackthorn", species **==** "Pyrus" **~** "pear"

))

**Summarize**

depletion.species_day_tree.sum <- depletion.species_day_tree **%>% group_by**(latin.name, date, tree) **%>% point_interval**(depletion.c, .point = median, .interval = hdci)

**Visualize**

“Mean depletion rate by species and tree. Point-intervals summarize the posterior in terms of the highest density interval of the median, with the inner and outer intervals representing 66% and 95% of the posterior, respectively. Note that *Prunus spinosa* (blackthorn) is not visualized. Since only a single bagged/open pair of flowers was sampled from each of the 27 blackthorn shrubs included in our study, tree-level depletion estimates for blackthorn have such wide uncertainty as to be minimally informative.”

*# Dropping P. spinosa because it makes the plot really crowded and we don't have good tree-level estima*

**ggplot**(**filter**(depletion.species_day_tree, latin.name **!=** "Prunus spinosa"),

**aes**(tree, y = depletion.c*****100, color = latin.name)) **+**

**stat_pointinterval**(point_interval = "median_hdci", position = **position_dodge**(width = 0.5)) **+ labs**(x = "Tree", y = "Depletion (%)", color = "Species") **+**

**scale_color_manual**(values = **c**("#F8766D", "#C77CFF", "#7CAE00")) **+ facet_wrap**(**~**date, scales = "free_x") **+**

**theme_lucid**() **+**

**theme**(panel.grid.minor = **element_blank**())

100

**2023−03−21**

**2023−03−22**

| **2023−03−20** | |
| --- | --- |
|  |  |
|  |  |
|  |  |
|  |  |

75

50

25

0

D03 D01 D02 D03 D01 D02 D03

**2023−03−29**

**2023−04−10**

100

Depletion (%)

75

50

25

0

D01 D04 D05 D06 C01 C02 D07 D08 C03 C04 D09 D10

#### Species

Prunus (Cerasus) Prunus domestica Pyrus communis

**2023−04−14**

100

**2023−04−19**

**2023−04−21**

75

50

25

0

D11 D12 P01 P02 P02 P03 P04

Tree

*# ggsave("../writing/plum_paper/figures/figS3.pdf", width = 9, height = 7) # ggsave("../writing/plum_paper/figures/figS3.png", width = 9, height = 7)*
